# Biotransformation of Bicyclic Halolactones with a Methyl Group in the Cyclohexane Ring into Hydroxylactones and Their Biological Activity

**DOI:** 10.3390/molecules21111453

**Published:** 2016-10-31

**Authors:** Katarzyna Wińska, Małgorzata Grabarczyk, Wanda Mączka, Barbara Żarowska, Gabriela Maciejewska, Katarzyna Dancewicz, Beata Gabryś, Antoni Szumny, Mirosław Anioł

**Affiliations:** 1 Department of Chemistry, Wrocław University of Environmental and Life Sciences, Norwida 25, 50-375 Wrocław, Poland; magrab@onet.pl (M.G.); wanda_m19@o2.pl (W.M.); antjasz@o2.pl (A.S.); miroslaw.aniol@up.wroc.pl (M.A.); 2 Department of Biotechnology and Food Microbiology, Wrocław University of Environmental and Life Sciences, Chełmońskiego 37/41, 51-630 Wrocław, Poland; barbara.zarowska@up.wroc.pl; 3 Faculty of Chemistry, Wroclaw University of Technology, Wybrzeze Wyspianskiego 27, 50-370 Wroclaw, Poland; gabriela.maciejewska@pwr.edu.pl; 4 Department of Botany and Ecology, University of Zielona Gora, Szafrana 1, 65-516 Zielona Gora, Poland; k.dancewicz@wnb.uz.zgora.pl (K.D.); b.gabrys@wnb.uz.zgora.pl (B.G.)

**Keywords:** lactones, hydrolytic dehalogenation, antimicrobial activity, deterrent activity

## Abstract

The aim of this study was the chemical synthesis of a series of halo- and unsaturated lactones, as well as their microbial transformation products. Finally some of their biological activities were assessed. Three bicyclic halolactones with a methyl group in the cyclohexane ring were obtained from the corresponding γ,δ-unsaturated ester during a two-step synthesis. These lactones were subjected to screening biotransformation using twenty two fungal strains. These strains were tested on their ability to transform halolactones into new hydroxylactones. Among the six strains able to catalyze hydrolytic dehalogenation, only two (*Fusarium equiseti*, AM22 and *Yarrowia lipolytica*, AM71) gave a product in a high yield. Moreover, one strain (*Penicillium wermiculatum*, AM30) introduced the hydroxy group on the cyclohexane ring without removing the halogen atom. The biological activity of five of the obtained lactones was tested. Some of these compounds exhibited growth inhibition against bacteria, yeasts and fungi and deterrent activity against peach-potato aphid.

## 1. Introduction

Due to their properties, chloro-organic compounds are used in many areas. They are very often applied as antifungal [1,2], antibacterial [3], antifidant [4,5], cytotoxic, and anticancer agents [6]. They are also used as organic solvents, refrigerants and media, dielectrics, pesticides, monomers for the production of plastic materials, and intermediates for organic synthesis [7]. Usually, these compounds are obtained by organic synthesis although they are increasingly being obtained from natural substances too. The compounds are obtained mainly from marine organisms such as red algae, corals or bacteria [8,9,10]. Longifoliosides A and B, which were isolated from *Veronica longifolia,* exhibit radical-scavenging activity against nitric oxide, superoxide, and 2,2-diphenyl-1-picrylhydrazyl radicals [11]. Halogened compounds could also be obtained by using the biotransformation method. Four antimicrobial halogenated compounds, bromomethyl- chlamydosporols A, B, chlamydosporol and fusarielin A, were isolated from a culture of *Fusarium tricintum*. These halogenated chlamydosporol analogues were obtained as products of the fermentation of a marine fungus. The transformation process was inducted by the addition of CaBr_2_ [12]. All the obtained compounds exhibited low antibacterial activity against *Staphylococcus aureus*, methicillin-resistant *S. aureus*, and multidrug-resistant *S. aureus*.

Isolated from the Australian ascidian *Aplidium caelestis* brominated natural products were tested against three mammalian cell lines (MCF-7, NFF, and MM96L), but they showed only minor cytotoxicity [13]. The bromotyrosine alkaloid psammaplysin was isolated from the Australian marine sponge *Hyattella* sp, along with psammaplysin F. These compounds were tested against two different strains of the parasite *Plasmodium falciparum* (Dd2 and 3D7). The tests showed that one of them, psammaplysin G, inhibited the chloroquine-resistant (Dd2) strain of *P. falciparum* by 98% [14].

Despite the interesting biological properties of halogenated organic compounds, in the 60s it was found that they may accumulate in soil or water without being broken down. At high concentrations, these compounds may have a negative impact on living organisms, endangering their health or life. For decades, researchers conducted experiments on the effects of waste on living organisms. It has been proven that halogenated organic compounds can cause endocrine system disorders in humans [15]. Therefore, effective methods for disposing of organic halo compounds have been the subject of research since the middle of the last century. One of the most popular methods is to transform them using filamentous fungi utilizing their ability for hydrolytic dehalogenation. Our team has been conducting studies dealing with halolactone transformations since 1998 [16]. In these studies we have obtained new compounds with interesting biological properties.

## 2. Results and Discussion

**Scheme 1:**
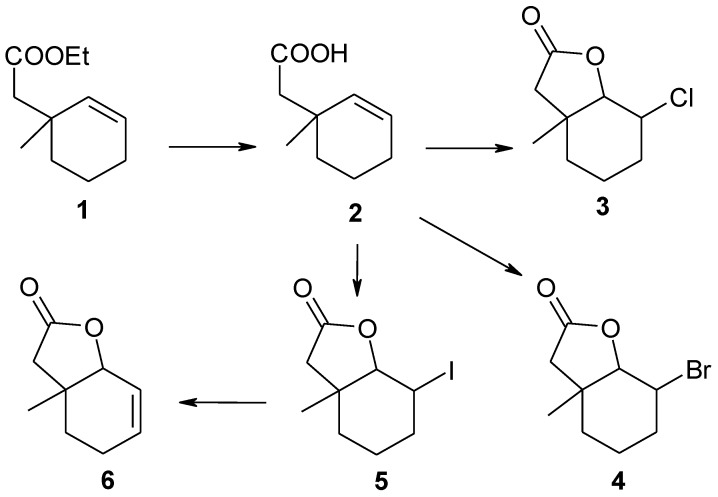
Synthesis of lactones **3**–**6**.

Substrates for biotransformation halolactones **3**–**5** were obtained during the two-step synthesis from the unsaturated ester **1**. Firstly, the ester was hydrolyzed to the γ,δ-unsaturated acid **2**. In the second step, this acid was transformed into the new chloro-**3**, bromo-**4**, and known iodolactone **5**. Iodolactone **5** further reacted with DBU giving the unsaturated lactone **6** (Scheme 1).

**Figure 1:**
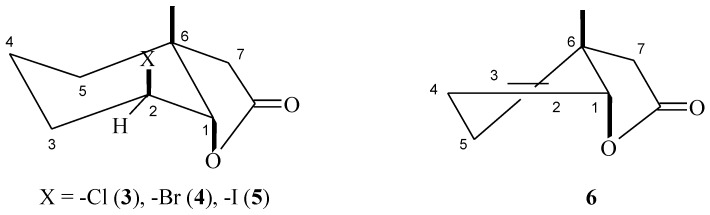
Structure of lactones **3**–**6**.

The structures of these compounds were established on the basis of their spectral data (^1^H-NMR, ^13^C-NMR, COSY, HMQC, and IR) and confirmed by HRMS analysis. In the IR spectra the absorption bands at 1780 (for **3**), 1772 (for **4**), 1776 (for **5**), and 1776 (for **6**) cm^−1^ confirmed the presence of the γ-lactone ring in the halolactone molecules and the unsaturated lactone. Analysis of the ^1^H-NMR spectra of halolactones allowed for the determination of the orientations of protons H-1 and H-2. The signal coming from proton H-1 appeared as a narrow singlet in the case of chloralactone **3** and for **4** and **5** it was a doublet with the small coupling constant (5.2 Hz for **4** and 6.1 Hz for **5**). Such shapes for these signals suggest their equatorial orientation and therefore an axial orientation of the C-O bond of the lactone ring. Also, a narrow multiplet coming from the H-2 protons indicated their equatorial orientation, which clearly indicated an axial position of the halogen atom. Such observations confirmed that the cyclohexane ring was in a chair conformation. If so, because the C-O bond of the lactone ring was in an axial position, the methyl group at carbon C-6 must be in an axial orientation. In the case of unsaturated lactone **6,** the signal coming from proton H-1 looked like a narrow multiplet and indicated its equatorial position. Wide multiplets coming from protons H-2 and H-3 suggested their axial orientation. (Figure 1).

**Scheme 2:**
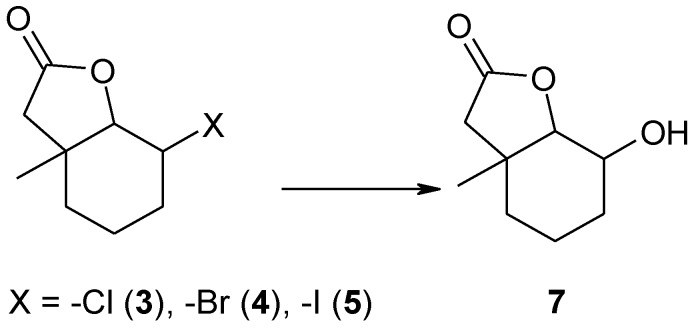
The expected biotransformation product **7** of halolactones **3**–**5**.

Our previous experiences indicated that in most cases halolactones subjected to biotransformation by filamentous fungi strains were converted into hydroxylactones. This reaction—hydrolytic dehalogenation–always proceeded according to a mechanism similar to a S_N_2 substitution. Independently, the structure of the substrate OH group was always introduced in an equatorial position. Taking this into account, it was interesting to see if in this case the fungal strains would be able to conduct the reaction of hydrolytic dehalogenation, giving new hydroxylactone **7** (Scheme 2). The fourth substrate–unsaturated lactone **6** was also tested for its ability to give any product.

Screening biotransformation was conducted on twenty two fungal strains. The progress of all of these transformations was monitored using standard techniques (TLC and GC). During the present research, one of the chosen microorganisms (*Penicillium vermiculatum* AM30) generated a different product than the expected one. Taking this observation into consideration, the effects of the screening biotransformation are presented in Table 1 and Table 2.

As it can be seen in the tables, the biotransformed halocompounds were transformed into hydroxycompounds **7**–**10** by some microorganisms. From the eight *Fusarium* strains, only *Fusarium equiseti* AM22 (Table 1, entry 7) proved to be a good catalyst, giving a product in high yield when bromolactone **4** and iodolactone **5** were used as substrates. The second strain which was able to transform these halolactones into hydroxylactone **7** in good yield was *Yarrowia lipolytica* AM71 (Table 1, entry 18). Some of the other strains (Table 1, entries 8, 9, 11, 12) were able to create only a small percentage of product.

**Scheme 3:**
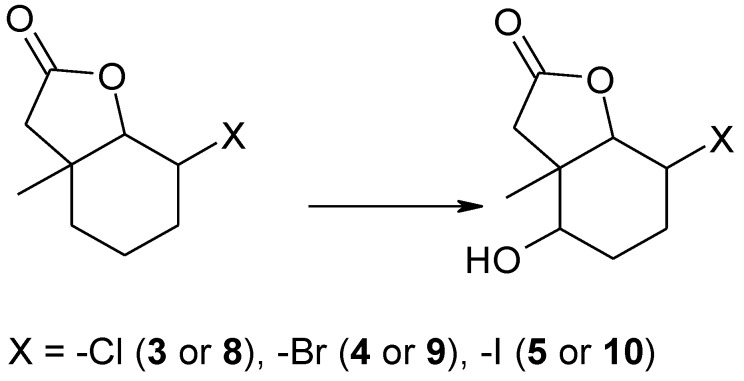
The products of biotransformation of halolactones **3**–**5** by *Penicillium vermiculatum* AM30.

It is worth noticing that *Penicillium camembertii* AM83 transformed only one of the halolactones. During the screening biotransformation it turned out that *Penicillium vermiculatum* AM30 strain (Table 2, entry 1) was able to produce different products than the other microorganisms. This was very surprising because till now we had not observed any products when this strain was used for biotransformation. It turned out that *P. vermiculatum* produced new hydroxyhalolactones **8**–**10**. These lactones were obtained as a product of hydroxylation on carbon C-5 without the removal of the halogen atom from carbon C-2 (Scheme 3).

As mentioned earlier, unsaturated lactone **6** was also checked for its ability to give any product. Unfortunately none of the tested microorganisms were able to convert this substrate.

**Figure 2:**
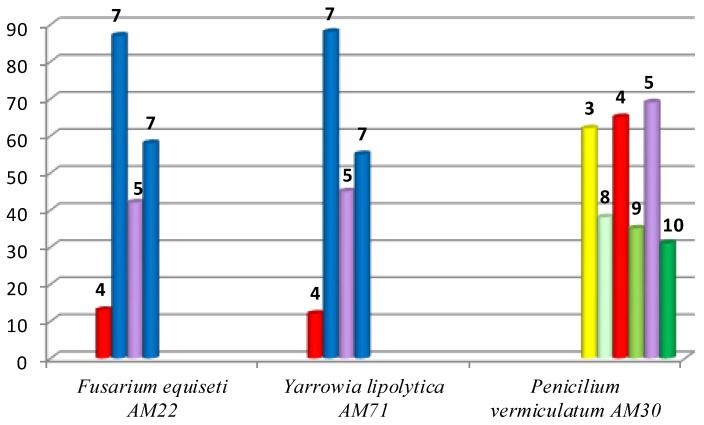
Results of preparative scale biotransformations carried out on halolactones **3**–**5** (by GC).

Since the aim of the present study was to obtain and characterize the structures of the biotransformation productsf—hydroxylactone **7** and also hydroxy-halolactones **8**–**10**—preparative scale biotransformations were performed in the next step. The best strains selected during the screening biotransformation were used for this purpose. The results of this are shown in Figure 2.

Two strains—*F. equiseti* and *Y. lipolytica—*were able to convert lactones **4**, **5** into the same product—hydroxylactone **7**. The better substrate was bromolactone **4**, which was transformed into a product with a very good degree of conversion (87% and 88%). In the case of iodolactone **5** the product was created with a lower conversion (55%–58%). The third strain *P. vermiculatum* led to hydroxyhalolactones with low efficiency, due to a narrow degree of conversion of the substrates (between 31% and 38%). For this reason it was only possible to check their chemical properties.

**Figure 3:**
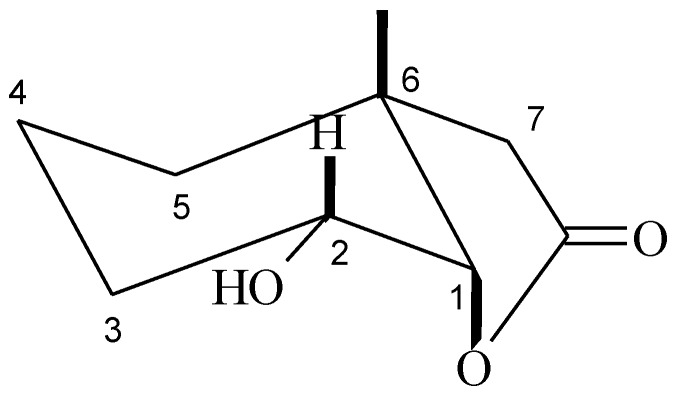
Structure of hydroxylactone **7**.

Analysis of ^1^H-NMR spectra of hydroxylactone **7** proved that the chair conformation of the cyclohexane ring was retained. The narrow signal coming from proton H-1 indicated its equatorial position. It means that the C-O bond of the lactone ring is located in an axial position, as in the substrates. The wide multiplet coming from proton H-2 suggested its axial orientation and consequently an equatorial position of the hydroxy group (Figure 3).

The hydrolytic dehalogenation reaction occurred similarly to a S_N_2 mechanism in every case. Moreover the hydroxy group was always introduced in the equatorial position in the cyclohexane ring. When a halogen atom occupied an equatorial position the simultaneous change of the conformation of the cyclohexane ring was observed during the reaction of hydrolytic dehalogenation [16,17]. When the halogen atoms occupied an axial position and the equatorial position was free, conformational change of the cyclohexane ring was not observed [18]. Because the halogen atom was in an axial position as described in the situation here, the conformation of the cyclohexane ring had not changed.

**Figure 4:**
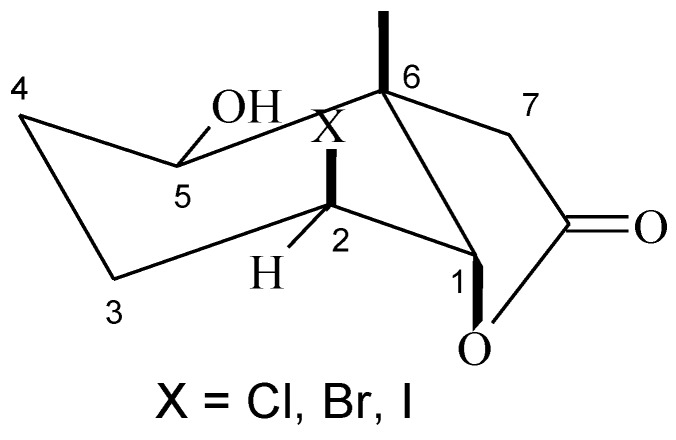
Structure of hydroxy-halolactone **8**–**10**.

Analysis of ^1^H-NMR spectra of the hydroxyhalolactones **8**–**10** proved that the halolactone ring structure was retained. Such a conclusion was evidenced by the narrow doublet coming from proton H-1 (*J* = 3.2 Hz for **8**, *J* = 3.5 Hz for **9**, and *J* = 4.0 Hz for **10**) and the wide multiplet coming from proton H-2, indicating their equatorial and axial position, respectively. Also the wide doublet of doublets (*J* = 10.7 Hz for **8**, *J* = 10.3 Hz for **9,** and *J* = 9.1 Hz for **10**) coming from the hydroxy group at carbon C-5 suggested its axial orientation and an equatorial position of the hydroxy group (Figure 4).

In the next step, the isolated yield, enantiospecificity, and optical purity of the hydroxylactone **7** and the hydroxyhalolactones **8**–**10** obtained during all the preparative biotransformations were determined. The results of shown in Table 3.

In all cases the (‒)-isomer of hydroxylactone **7** was formed preferentially. When bromolactone **4** was used as a substrate**,** the enantiomeric excesses did not exceed 11%. When hydroxylactone **7** was created from iodolactone **5** these values were much better, amounting to 52.9% and 27%. In the case of hydroxyhalolactones **8**–**10** the (+)-isomer was preferentially created, but the enantiomeric excesses did not exceed 7.6%.

Another aim in this research work was the verification of the biological properties of the obtained compounds. The obtained synthetic halolactones **3**–**6**, as well as the obtained microbiologically formed hydroxylactone **7**, were examined for their ability to inhibit the growth of some bacteria, yeast, and fungal strains. Statistical analysis was performed for the results of microbiological tests. Calculations were made for each of the strains separately. Control and further compounds were treated as test variability. The underlying assumption was the null hypothesis that the mean for the control and individual substances do not differ. The hypothesis was verified by a F-Senedecora test at the significance level of *p* = 0.01. It has been shown that for each strain averages are significantly different. Then Dunnett’s test was checked, in which means are significantly different from the average for the control. The results of this test are presented in Table 4.

The group of microorganisms exhibiting the greatest growth-inhibition by the test compounds were bacteria. In the case of the *Escherichia coli* strain, all lactones caused inhibition of the bacterial growth. This is a very interesting result, because so far the compounds which tested by us were relatively inactive to this strain. Lactones **3**, **4,** and **6** completely inhibited the growth of the *Staphylococcus aureus* strain. Compounds **5** and **7** also affected the growth of this strain of bacteria causing considerable extension phase adaptive (20–36 h) and a slight increase in the biomass expressed as ΔOD in the range of 0.37–0.62. It is worth noting that in the culture of the *Pseudomonas fluorescens* strain, conducted in the presence of **5**, diauxic growth was observed. The same effect was observed earlier for the *E. coli* strain and the *C. albicans* strain [19]. This phenomenon was also reported by other authors [20,21].

In the case of the *C. albicans* strain, lactone **7** stimulated the growth of these yeasts resulting in the growth of biomass, ΔOD was equal to 1.99, whereas the control yeast culture′s ΔOD value was 1.14. A similar effect of the stimulation of growth occurred in the case of a strain of *Fusarium avenaceum*. In this case compounds **6** and **7** produced an increase in the biomass of 0.62–0.69 compared to the control culture. Lactone **5** inhibited growth in the *Fusarium avenaceum* strain. Lactone **4** completely inhibited the growth of the *Fusarium avenaceum*. Other biological tests were conducted to determine the deterrent activity of lactones **3**–**7** against peach-potato aphid *Myzus persicae*. The results of this experiment are presented in Table 5.

**Figure 5:**
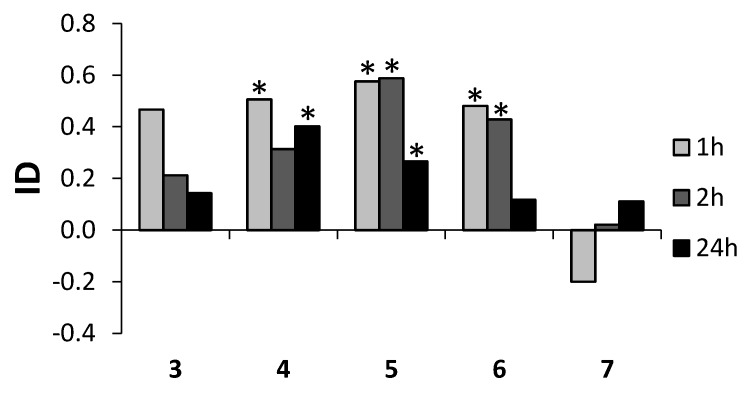
Effect of lactones **3**–**7** on settling preferences of *Myzus persicae*in the choice test. ID—index of deterrence, * *p* < 0.05 (according to statistical analysis using Student *t*-test) (STATISTICA (data analysis software system), version 10, 2014, StatSoft, Inc., Tulsa, OK, USA, www.statsoft.com).

Among the tested lactones, bromolactone **4** and iodolactone **5** showed high and persistent deterrent properties. The application of these compounds caused the avoidance of plants by aphids as soon as 1 h after exposure and the effect persisted at least until the 24th hour of the experiment. However, the level of activity of **5** tended to subside over the course of time. The other lactones, chlorolactone **3** and the unsaturated lactone **6** also showed deterrent capacity. The relatively strong deterrent effect of **6** ceased before the 24th hour after application. Hydroxylactone **7** initially expressed a tendency to attract aphids, but this tendency reversed over the course of time (Table 5, Figure 5). The changes in the deterrence potency of various low molecular terpenoid lactones are typical for this group of compounds. The switch from attractant to deterrent properties or otherwise was found in the case of piperitone-derived saturated lactones, halolactones and hydroxylactones [22,23]. Literature data [24] suggest that the high enantiomeric excess affected the deterrent activity of the aphids. The halolactones **3**–**5** presented here were the racemic mixtures. In the case of hydroxylactone **7**, *ee* values were not very high. Because of that, it is impossible to determine any influence of the enantiomeric excesses of these lactones on the deterrent activity of the aphids.

## 3. Experimental Section

### 3.1. General Information

TLC was performed on silica gel-coated aluminium plates (DC-Alufolien Kieselgel 60 F254, Merck: Darmstadt, Germany) using a mixture of hexane and acetone in various ratios. Preparative column chromatography was performed on the silica gel (Kieselgel 60, 230–400 mesh ASTM, Merck) with a mixture of hexane and acetone (for unsaturated lactone **6** and biotransformation products hexane-acetone 3:1, for halolactones **3**–**5** hexane-acetone 6:1) as eluents. GC analysis was carried out on an Agilent Technologies 6890N instrument (Varian, Agilent Technologies, Santa Clara, CA, USA) instrument using a DB-17 column (cross-linked methyl silicone gum, 30 m × 0.32 mm × 0.25 µm). The enantiomeric compositions of the products obtained during the biotransformation were determined by GC analysis using the chiral column CP-cyclodextrin-B-110 (30 m × 0.25 mm × 0.25 µm) (Supelco, Bellefonte, PA, USA) under the following conditions: injector 200 °C, detector (FID) 200 °C, column temperature 120 °C, hold 105 min; ramp 120–135 °C at a rate 1 °C/min, ramp 135–200 °C at a rate 20 °C/min and hold 1 min at 200 °C (for hydroxylactone **7** Rt: (+) 115 min, (−) 116 min ) and injector 200 °C, detector (FID) 200 °C, column temperature 140 °C, ramp 140–160 °C at a rate 0.5 °C/min, ramp 160–200 °C at a rate 20 °C/min and hold 1 min at 200 °C (for hydroxyhalolactones: **8** Rt: (−) 36 min, (+) 38 min; **9** Rt: (−) 27 min, (+) 29 min; **10** Rt: (−) 30 min, (+) 32 min).

The molar masses of the obtained compounds were confirmed by high resolution mass spectrometry analysis using a Waters LCT Premier XE instrument (ESI ionisation) (Waters Division, Milford, MA, USA).

^1^H-NMR spectra were recorded in a CDCl_3_ solution on a Bruker Avance™ 600 MHz spectrometer (Bruker, Billerica, MA, USA). Chemical shifts are reported in reference to the residual solvent signal (δ_H_ = 7.26). IR spectra were recorded on a IR300 FT-IR spectrometer (Thermo-Nicolet, Waltham, MA, USA). Optical rotations were determined on a P-2000 polarimeter (Jasco, Easton, PA, USA) in chloroform solutions, with concentrations denoted in g/100 mL. The melting points were determined on a Boetius apparatus. The refractive index was measured on a Carl Zeiss Abbe and Pulfrich refractometer (Jena, Germany).

### 3.2. Synthesis of Substrates

Unsaturated ester **1** was hydrolyzed into the known [25] γ,δ-unsaturated acid **2** according to the known method [26]. This acid was then converted into the new chloro-**3**, bromo-**4**, and known [25] iodolactone **5** according to the procedure described earlier [27]. Iodolactone **5** was then used to obtain unsaturated lactone **6**. Here we present the synthesis and spectral data of compounds **3**–**6**, more Data can be found in the Supplementary Materials. This data will be useful in studying the relationship between the structures of the substrates and their interaction with microorganisms.

*2-Chloro-6-methyl-9-oxabicyclo[4.3.0]nonan-8-one* (**3**). Chlorolactonization of acid **2** (2.5 g, 0.016 mol), according to the procedure of [26] gave 1.6 g (76%) of chlorolactone **3** with the following physical and spectral properties: m.p. = 42–43 °C, ^1^H-NMR (600 MHz, CDCl_3_): 1.34 (s, 3H, CH_3_-9), 1.49–1.51 (m, 1H, one of CH_2_-4), 1.53–1.57 (m, 1H, one of CH_2_-5), 1.59–1.63 (m, 1H, one of CH_2_-5), 1.83–1.85 (m, 2H, one of CH_2_-4 and one of CH_2_-3), 2.05–2.09 (m, 1H, one of CH_2_-3), 2.31 (d, *J* = 16.9 Hz, 1H, one of CH_2_-7), 2.42 (d, *J* = 16.9, 1H, one of CH_2_-7), 4.18 (m, 1H, H-2), 4.22 (m, 1H, H-1), ^13^C-NMR (151 MHz, CDCl_3_): 17.62 (C-4), 25.36 (C-9), 30.54 (C-3), 32.92 (C-5), 39.42 (C-1), 42.95 (C-7), 57.02 (C-2), 86.84 (C-1), 175.08 (C-8), IR (KBr, cm^−1^): 2958, 1780, 1449, 1159, 1022, 993, ESIHRMS: calcd for C_9_H_13_ClO_2_, *m*/*z* 189.0677 [M + H]^+^, found 189.0682.

*2-Bromo-6-methyl-9-oxabicyclo[4.3.0]nonan-8-one* (**4**). After the bromolactonization of 1.7 g (0.011 mol) of acid **3**, according to the known method [26], 1.6 g (61%) of bromolactone **4** was obtained. The physical and spectral data of this product are as follows: m.p. = 49–50 °C, ^1^H-NMR (600 MHz, CDCl_3_): 1.36 (s, 3H, CH_3_-9), 1.51–1.54 (m, 1H, one of CH_2_-4), 1.54–1.56 (m, 1H, one of CH_2_-5), 1.63–1.67 (m, 1H, one of CH_2_-5), 1.83–1.86 (m, 1H, one of CH_2_-4), 1.91–1.96 (m, 1H, one of CH_2_-3), 2.16–2.20 (m, 1H, one of CH_2_-3), 2.28 (d, *J* = 16.9 Hz, 1H, one of CH_2_-7), 2.46 (d, *J* = 16.9, 1H, one of CH_2_-7), 4.20 (m, 1H, H-2), 4.36 (d, *J* = 5.2 Hz, 1H, H-1), ^13^C-NMR (151 MHz, CDCl_3_): 18.95 (C-4), 26.10 (C-9), 31.67 (C-3), 32.97 (C-5), 39.86 (C-1), 42.41 (C-7), 48.58 (C-2), 87.42 (C-1), 174.92 (C-8), IR (KBr, cm^−1^): 2958, 1772, 1448, 1156, 1019, 993, ESIHRMS: calcd for C_9_H_13_BrO_2_, *m*/*z* 233.0172 (M + H)^+^, found 233.0177.

*2-Iodo-6-methyl-9-oxabicyclo[4.3.0]nonan-8-one* (**5**). Iodolactonization of 1.8 g (0.012 mol) of acid **2**, according to the known procedure [26] gave 1.9 g (58%) of iodolactone **5** with the following physical and spectral properties: m.p. = 60–62 °C, ^1^H-NMR (600 MHz, CDCl_3_): 1.37 (s, 3H, CH_3_-9), 1.50–1.58 (m, 2H, one of CH_2_-4 and one of CH_2_-5), 1.70–1.75 (m, 2H, one of CH_2_-4, and one of CH_2_-5), 1.97–2.02 (m, 1H, one of CH_2_-3), 2.18–2.19 (m, 1H, one of CH_2_-3), 2.21 (d, *J* = 16.9 Hz, 1H, one of CH_2_-7), 2.53 (d, *J* = 16.9, 1H, one of CH_2_-7), 4.21 (m, 1H, H-2), 4.46 (d, *J* = 6.1 Hz, 1H, H-1), ^13^C-NMR (151 MHz, CDCl_3_): 21.13 (C-4), 25.97 (C-2), 27.21 (C-9), 33.05 (C-5), 34.21 (C-3), 40.15 (C-6), 41.36 (C-7), 89.25 (C-1), 174.73 (C-8), IR (KBr, cm^−1^): 2956, 1776, 1457, 1149, 987, ESIHRMS: calcd for C_9_H_13_IO_2_, *m*/*z* 281.0033 (M + H)^+^, found 281.0038.

*6-Methyl-9-oxabicyclo[4.3.0]non-2-en-8-one* (**6**). Iodolactone **5** (0.25 g, 0.0009 mol) was heated with DBU in toluene for three hours. After standard purification was obtained 0.125 g (92%) of unsaturated lactone 7 with the following physical and spectral properties: colourless oil, ^1^H-NMR (600 MHz, CDCl3): 1.19 (s, 3H, CH3-9), 1.56 (dt, *J* = 13.6 and 5.5 Hz, 1H, one of CH2-5), 1.67 (ddd, *J* = 13.6, 7.5 and 6.0 Hz, 1H, one of CH2-5), 2.13–2.15 (m, 2H, CH2-4), 2.34 (d, *J* = 17.1 Hz, 1H, one of CH2-7), 2.47 (d, *J* = 17.1, 1H, one of CH2-7), 4.30 (m, 1H, H-1), 5.81–5.84 (m, 1H, H-2), 6.06–6.10 (m, 1H, H-3), ^13^C-NMR (151 MHz, CDCl3): 21.70 (C-4), 23.37 (C-9), 29.38 (C-5), 37.11 (C-6), 41.97 (C-7), 81.08 (C-1), 122.89 (C-2), 133.05 (C-3), 176.28 (C-8), IR (KBr, cm-1): 2963, 1776, 1211, 1154, 989, ESIHRMS: calcd for C_9_H_12_O_2_, *m*/*z* 153.0910 (M + H)^+^, found 153.0916.

### 3.3. Microorganisms

The fungal and yeast strains used for biotransformation were obtained from the collection of the Department of Chemistry, Wrocław University of Environmental and Life Sciences. They were: *Fusarium culmorum* AM10, *Fusarium avenaceum* AM11, *Fusarium oxysporum* AM13, *Fusarium tricinctum* AM16, *Fusarium semitectum* AM20, *Fusarium oxysporum* AM21, *Fusarium equiseti* AM22, *Fusarium solani* AM203, *Penicillium vermiculatum* AM30, *Penicillium camembertii* AM83, *Penicillium chermesinum* AM113, *Penicillium frequentans* AM351, *Absidia cylindrospora* AM336, *Absidia corerulea* AM93, *Aspergillus ochraceus* AM456, *Aspergillus wenthi* AM413, *Syncephalastrum racemosum* AM105, *Pleurotus ostreatus* AM482, *Pleurotus ostreatus* AM600, *Mucor hiemalis* AM450, *Yarrovia lipolytica* AM71, and *Rhodotorula marina* AM77. The fungal and yeast strains were cultivated on Sabouraud agar.

#### 3.3.1. Screening Procedure

The fungal strains were cultivated in 300 mL Erlenmayer flasks, containing 100 mL of the medium consisting of glucose (3 g) and peptobac (1 g). After three days when the culture was grown, 10 mg of the substrate dissolved in 1 mL of acetone was added to each flask. The incubation of the shaken cultures with the substrate was continued for 7 days. After 3, 5, and 7 days of incubation, the medium contained the unreacted substrate, and the product and mycelium were extracted with dichloromethane (15 mL) and analyzed by GC (DB-17 column).

#### 3.3.2. Preparative Biotransformation

Reactions were conducted in ten 300 mL Erlenmayer flasks containing 3-day cultures of fungal or yeast strain (prepared similarly as described above). In this case, 100 mg of substrate was dissolved in 10 mL acetone and added to ten flasks. After 7 days the post-reaction mixture was extracted with dichloromethane (3 × 40 mL). The combined organic fractions were dried over anhydrous magnesium sulphate and the solvent was evaporated in vacuo. Pure product was separated from the unreacted substrate and the metabolites of the fungi or yeast by using column chromatography (silica gel, hexane: acetone 3:1). As an effect of these reactions, four products were obtained, more Data can be found in the Supplementary Materials.

*2-Hydroxy-6-methyl-9-oxabicyclo[4.3.0]nonan-8-one* (**7**): m.p. = 68–69 °C, ^1^H-NMR (600 MHz, CDCl_3_): 1.21 (s, 3H, CH_3_-9), 1.42–1.47 (m, 2H, one of CH_2_-4 and one of CH_2_-5), 1.57–1.59 (m, 1H, one of CH_2_-3), 1.71–1.74 (m, 1H, one of CH_2_-4), 1.81–1.84 (m, 1H, one of CH_2_-3), 2.12 (m, 1H, OH), 2.37 (d, *J* = 16.5 Hz, 1H, one of CH_2_-7), 2.45 (d, *J* = 16.5, 1H, one of CH_2_-7), 3.84 (dt, *J* = 10.6 and 4.0 Hz, 1H, H-2), 4.24 (d, *J* = 3.3 Hz, 1H, H-1), ^13^C-NMR (151 MHz, CDCl_3_): 19.18 (C-4), 22.32 (C-9), 28.99 (C-3), 32.46 (C-5), 40.10 (C-6), 45.13 (C-7), 67.72 (C-2), 86.12 (C-1), 176.07 (C-8), IR (KBr, cm^−1^): 3352, 2938, 1773, 1458, 1201, 1065, 988, ESIHRMS: calcd for C_9_H_14_O_3_, *m*/*z* 171.1016 (M + H)^+^, found 171.1021.

*2-Chloro-5-hydroxy-6-methyl-9-oxabicyclo[4.3.0]nonan-8-one* (**8**): colourless oil, ^1^H-NMR (600 MHz, CDCl_3_): 1.37 (s, 3H, CH_3_-9), 1.73 (ddd, *J* = 12.7, 8.1 and 4.0 Hz, 1H, one of CH_2_-4), 1.98–2.04 (m, 1H, one of CH_2_-4), 2.12–2.15 (m, 2H, CH_2_-3), 2.38 (d, *J* = 17.2 Hz, 1H, one of CH_2_-7), 2.79 (d, *J* = 17.2, 1H, one of CH_2_-7), 3.63 (dd, *J* = 10.7 and 3.8 Hz, 1H, H-5), 4.34–4.36 (m, 1H, H-2), 4.46 (d, *J* = 3.2 Hz, 1H, H-1), ^13^C-NMR (151 MHz, CDCl_3_): 17.07 (C-9), 24.70 (C-4), 27.84 (C-3), 41.94 (C-7), 43.69 (C-6), 53.99 (C-2), 70.79 (C-5), 86.77 (C-1), 174.16 (C-8), IR (KBr, cm^−1^): 3434, 2954, 1759, 1437, 1073, 999, ESIHRMS: calcd for C_9_H_13_ClO_3_, *m*/*z* 205.0626 (M + H)^+^, found 205.0639.

*2-Bromo-5-hydroxy-6-methyl-9-oxabicyclo[4.3.0]nonan-8-one* (**9**): colourless oil, ^1^H-NMR (600 MHz, CDCl_3_): 1.41 (s, 3H, CH_3_-9), 1.75 (ddd, *J* = 13.0, 8.4 and 4.2 Hz, 1H, one of CH_2_-4), 1.99–2.04 (m, 1H, one of CH_2_-4), 2.20–2.22 (m, 2H, CH_2_-3), 2.36 (d, *J* = 17.2 Hz, 1H, one of CH_2_-7), 2.79 (d, *J* = 17.2, 1H, one of CH_2_-7), 3.65 (dd, *J* = 10.3 and 3.0 Hz, 1H, H-5), 4.37–4.39 (m, 1H, H-2), 4.58 (d, *J* = 3.5 Hz, 1H, H-1), ^13^C-NMR (151 MHz, CDCl_3_): 17.95 (C-9), 25.68 (C-4), 28.49 (C-3), 41.82 (C-7), 44.00 (C-6), 45.21 (C-2), 70.68 (C-5), 87.22 (C-1), 174.09 (C-8), IR (KBr, cm^−1^): 3427, 2953, 1772, 1434, 1220, 1074, 992, ESIHRMS: calcd for C_9_H_13_BrO_3_, *m*/*z* 249.0121 (M + H)^+^, found 249.0130.

*5-Hydroxy-2-iodo-6-methyl-9-oxabicyclo[4.3.0]nonan-8-one* (**10**): m.p. = 58–59 °C, ^1^H-NMR (600 MHz, CDCl_3_): 1.47 (s, 3H, CH_3_-9), 1.76–1.80 (m, 1H, one of CH_2_-4), 1.91–1.97 (m, 1H, one of CH_2_-4), 2.12–2.17 (m, 1H, one of CH_2_-3), 2.25–2.29 (m, 1H, one of CH_2_-3), 2.32 (d, *J* = 17.1 Hz, 1H, one of CH_2_-7), 2.45 (d, *J* = 17.1, 1H, one of CH_2_-7), 3.71 (dd, *J* = 9.6 and 3.6 Hz, 1H, H-2), 4.44 (dd, *J* = 9.1 and 4.4 Hz, 1H, one of CH_2_-5), 4.69 (d, *J* = 4.0 Hz, 1H, H-1), ^13^C-NMR (151 MHz, CDCl_3_): 19.09 (C-9), 22.53 (C-5), 28.03 (C-4), 30.64 (C-3), 42.00 (C-7), 44.77 (C-6), 71.10 (C-2), 89.20 (C-1), 174.16 (C-8), IR (KBr, cm^−1^): 3427, 2929, 1754, 1437, 1224, 1066, 977, ESIHRMS: calcd for C_9_H_13_IO_3_, *m*/*z* 296.9982 (M + H)^+^, found 296.9988.

### 3.4. Bioassay Tests on Microorganisms

In this step the following bacteria strains were used: *Escherichia coli* C1, *Staphylococcus aureus*, *Bacillus subtilis* B5, yeast strains: *Candida albicans* KL-1 and filamentous fungi strains: *Aspergillus niger* XP, *Fusarium linii* 3A, *Penicillium* sp., *Alternaria* sp. The bacteria strains were obtained from the collection of the Department of Biotechnology and Food Microbiology, Wrocław University of Environmental and Life Sciences. The test were performed in the automated Bioscreen C system (Automated Growth Curve Analysis System, Lab Systems, Helsinki, Finland) with a procedure analogous to the one described before [15].

### 3.5. Aphid Settling Bioassays

The test were performed according to a procedure analogous to the one described before [17].

## 4. Conclusions

Four lactones were obtained from an γ,δ-unsaturated carboxylic acid via Claisen rearrangements. The four compounds were subjected to a screening biotransformation using twenty fungal strains and two yeast strains. Only some of them, namely *F. equiseti* AM22, *F. solani* AM203, *P. camembertii* AM83, *A. cylindrospora* AM336, *S. racemosum* AM105, and *Y. lipolytica* AM71, were able produce a hydrolytic dehalogenation of the halolactones, giving as a product the hydroxylactone **7**. Additionally the *P. vermiculatum* AM30 strain gave other products—the new hydroxyhalolactones **8**–**10**. Hydroxylactone **7** was preferentially formed as a (‒)-isomer, while in the case of hydroxy-halolactones **8**–**10**, a (+)-isomer was preferred. The biological tests proved that lactones mostly inhibit bacteria strains (*E. coli* and *S. aureus*). A similar effect was observed for the *F. avenaceum* strain (for lactones **4** and **5**). On the other hand, in the case of the *C. albicans* and the *F. avenaceum* strains, the tested lactones **7** and **6**, **7** caused a stimulation of biomass growth. Biological assays conducted on *M. persicae* showed that lactones **4** and **5** possesses the best deterrent activity. The unsaturated lactone **6** also showed feeding deterrent activity, but the strongest effect only lasted for two hours. Unexpectedly, hydroxylactone **7** was an attractant during the first hour and then its activity changed to a weak deterrent.

## Figures and Tables

**Table 1:** Results of the screening biotransformation of lactones **3**, **4**, and **5** after 7 days of incubation (in % according to GC).

Entry	Microorganism	Screening Biotransformation/%					
		3	7	4	7	5	7
1	*Fusarium culmorum* AM10	100	-	100	-	100	-
2	*Fusarium avenaceum* AM11	100	-	100	-	100	-
3	*Fusarium oxysporum* AM13	100	-	100	-	100	-
4	*Fusarium tricinctum* AM16	100	-	100	-	100	-
5	*Fusarium semitectum* AM20	100	-	100	-	100	-
6	*Fusarium oxysporum* AM21	100	-	100	-	100	-
7	*Fusarium equiseti* AM22	65.4	34.6	3.3	**96.7**	40.0	**60.0**
8	*Fusarium solani* AM203	86.1	13.9	76.6	23.4	74.7	25.3
9	*Syncephalastrum racemosum* AM105	100	-	74.0	26.0	69.9	30.1
10	*Absidia coerulea* AM93	100	-	100	-	100	-
11	*Absidia cylindrospora* AM336	100	-	75.0	25.0	86.0	14.0
12	*Penicillium camembertii* AM83	85.3	14.7	100	-	100	-
13	*Penicillium chermesinum* AM113	100	-	100	-	100	-
14	*Penicillium frequentans* AM351	100	-	100	-	100	-
13	*Aspergillus wenthi* AM413	100	-	100	-	100	-
14	*Aspergillus ochraceus* AM456	100	-	100	-	100	-
15	*Pleurotus ostreatus* AM482	100	-	100	-	100	-
16	*Pleurotus ostreatus* AM600	100	-	100	-	100	-
17	*Mucor hiemalis* AM450	100	-	100	-	100	-
18	*Yarrowia lipolytica* AM71	75.5	27.4	13.3	**86.7**	40.7	**59.3**
19	*Rhodotorula marina* AM77	100	-	100	-	100	-

Numbers in bold mean they are the best results of biotransformation.

**Table 2:** Results of the screening biotransformation of lactones **3**, **4**, and **5** after 7 days of incubation (in % according to GC).

Entry	Microorganism	Screening Biotransformation/%					
		3	8	4	9	5	10
1	*Penicillium vermiculatum* AM30	69.7	30.3	79.1	20.9	65.4	34.6

**Table 3:** Results of the preparative biotransformations of lactones **3**–**5**.

Substrate	Strain	Product	Isolated Yield g/%	Enatiomeric Excess (%)	Optical Rotation ${\left[\alpha \right]}_{D}^{20}$	Concentration in CH_3_Cl (g/100 mL)
**3**	*P. vermiculatum* AM30	**8**	0.0098/9.0	7.6	+5.404	0.48
**4**	*F. equiseti* AM22	**7**	0.028/38.4	10.7	−6.762	0.56
**4**	*Y. lipolytica* AM71	**7**	0.021/28.8	5.8	−6.244	0.62
**4**	*P. vermiculatum* AM30	**9**	0.0114/10.7	1.6	+0.645	0.78
**5**	*F. equiseti* AM22	**7**	0.016/26.3	27.0	−17.886	0.44
**5**	*Y. lipolytica* AM71	**7**	0.024/40.2	52.9	−31.214	0.51
**5**	*P. vermiculatum* AM30	**10**	0.015/14.2	5.5	+4.108	0.67

Optical rotation was measured for 589 nm and temperature 20 °C.

**Table 4:** Antimicrobial activity of lactones **3**–**7**. Statistics on a completely randomized design were determined using the one-way analysis of variance (ANOVA) procedure at a level of significance set at *p* = 0.010. A comparison of average growth OD microorganisms relative to the control by Dunnett’s test. Underlined numbers mean the average was significantly different from control.

Strain		*E. coli*	*S. aureus*	*P. fluorescens*	*C. albicans*	*F. avenaceum*	*A. niger*
Control	Lag-phase [h]	5	16	8	14.5	15.5	7.5
	ΔOD ± SD	1.29 ± 0.07	1.09 ± 0.05	1.36 ± 0.07	1.14 ± 0.05	1.18 ± 0.04	1.94 ± 00.6
**3**	Lag-phase [h]	9	-	14.5	21	35	13
	ΔOD ± SD	0.29 ± 0.04	0	1.05 ± 0.06	1.11 ± 0.04	1.40 ± 0.05	1.48 ± 0.05
**4**	Lag-phase [h]	18	-	16	29	-	13
	ΔOD ± SD	0.43 ± 0.05	0	1.08 ± 0.04	1.18 ± 0.04	0	1.02 ± 0.04
**5**	Lag-phase [h]	9	23	13	15	38	9
	ΔOD ± SD	0.32 ± 0.03	0.37 ± 0.04	0.95 ± 0.03	0.95 ± 0.03	0.71 ± 0.04	1.97 ± 0.05
**6**	Lag-phase [h]	9	-	14	21	32.5	11.5
	ΔOD ± SD	0.27 ± 0.03	0	1.32 ± 0.02	0.77 ± 0.02	1.80 ± 0.06	1.33 ± 0.07
**7**	Lag-phase [h]	5	36	12	19.5	14.5	7.5
	ΔOD ± SD	0.57 ± 0.05	0.62 ± 0.04	1.15 ± 0.04	1.99 ± 0.06	1.87 ± 0.07	1.94 ± 0.06

OD—Optical Density; OD was measured for λ 550 nm; SD—Standard Deviation.

**Table 5:** Deterrent activity of lactones **3**–**7**. Numbers for C (= control) and T (= treated) represent the mean number (±SE) of aphids that settled on the control or the treated leaf (choice-test) 1 h, 2 h, and 24 h after exposure. *p*—significance level (Student *t*-test)—significant differences (*p* < 0.05) between the number of aphids settled on either leaf are underlined.

	1 h			2 h			24 h		
	C	T	*p*	C	T	*p*	C	T	*p*
**3**	2.8 (±0.8)	1.0 (±0.3)	0.0615	2.5 (±0.6)	1.6 (±0.3)	0.2189	6.0 (±1.0)	4.5 (±1.5)	0.4168
**4**	7.6 (±1.1)	2.5 (±0.5)	0.0007	5.5 (±1.7)	2.9 (±0.7)	0.1791	8.5 (±1.5)	3.6 (±0.8)	0.0116
**5**	3.3 (±0.8)	0.9 (±0.3)	0.0106	3.4 (±0.5)	0.9 (±0.5)	0.0028	6.3 (±0.9)	3.6 (±0.8)	0.0470
**6**	9.6 (±1.3)	3.4 (±1.3)	0.0035	10.6 (±1.5)	4.3 (±1.3)	0.0067	7.8 (±1.8)	6.1 (±1.5)	0.4972
**7**	1.8 (±0.7)	2.6 (±1.0)	0.4951	3.0 (±1.0)	2.9 (±0.9)	0.9285	5.6 (±0.8)	4.5 (±1.0)	0.3996

## Supplementary Materials

The following are available online at http://www.mdpi.com/1420-3049/21/11/1453/s1.
