# Supplementary material for: Biotransformation of Bicyclic Halolactones with a Methyl Group in the Cyclohexane Ring into Hydroxylactones and Their Biological Activity
Source: Molecules. 2016 Oct 31;21(11):1453. doi: 10.3390/molecules21111453 (PMC6273801; doi:10.3390/molecules21111453)
Supplement: Supplementary file 1 [file molecules-21-01453-s001.pdf]

Supplementary Materials: Biotransformation of Bicyclic Halolactones with the Methyl Group in Cyclohexane Ring into Hydroxylactones and Their Biological Activity

Katarzyna Wińska, Małgorzata Grabarczyk, Wanda Mączka, Barbara Żarowska, Gabriela Maciejewska, Katarzyna Dancewicz, Beata Gabryś, Antoni Szumny and Mirosław Anioł

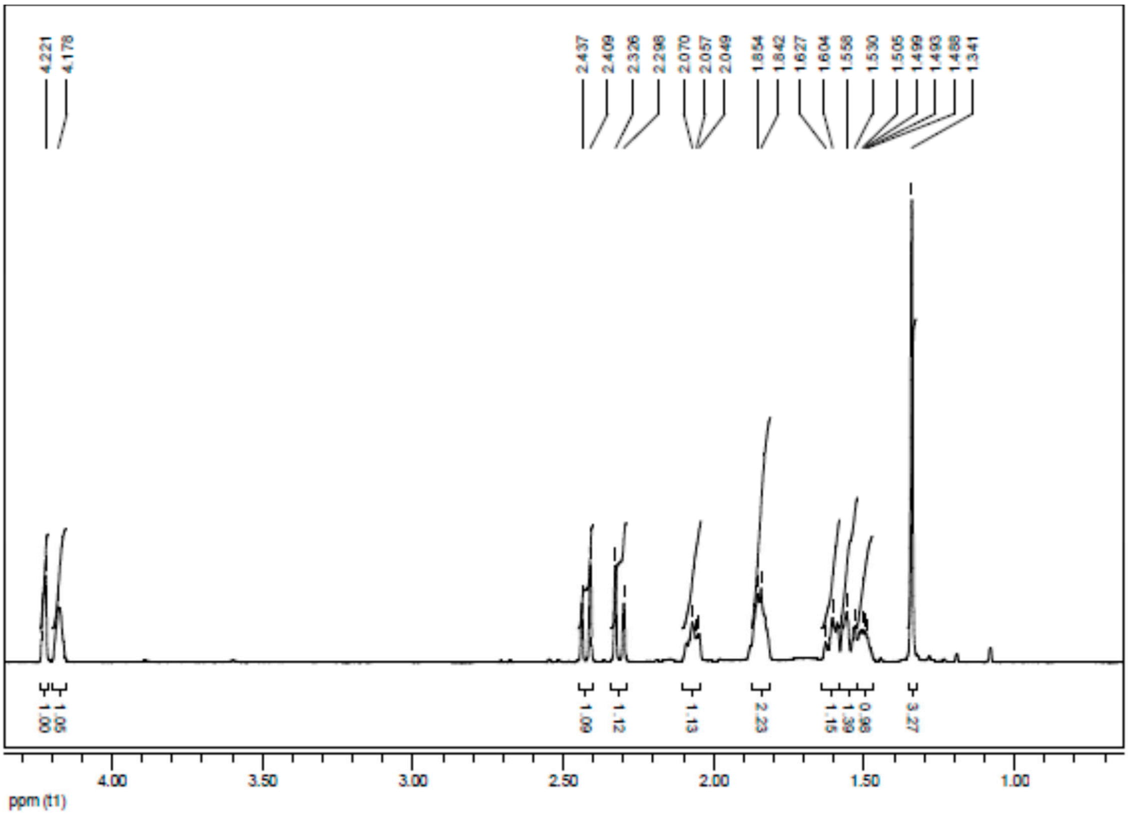

Figure S1. ^{1}H-NMR (600 MHz, $CDCl_3$) spectrum of chlorolactone **3**.

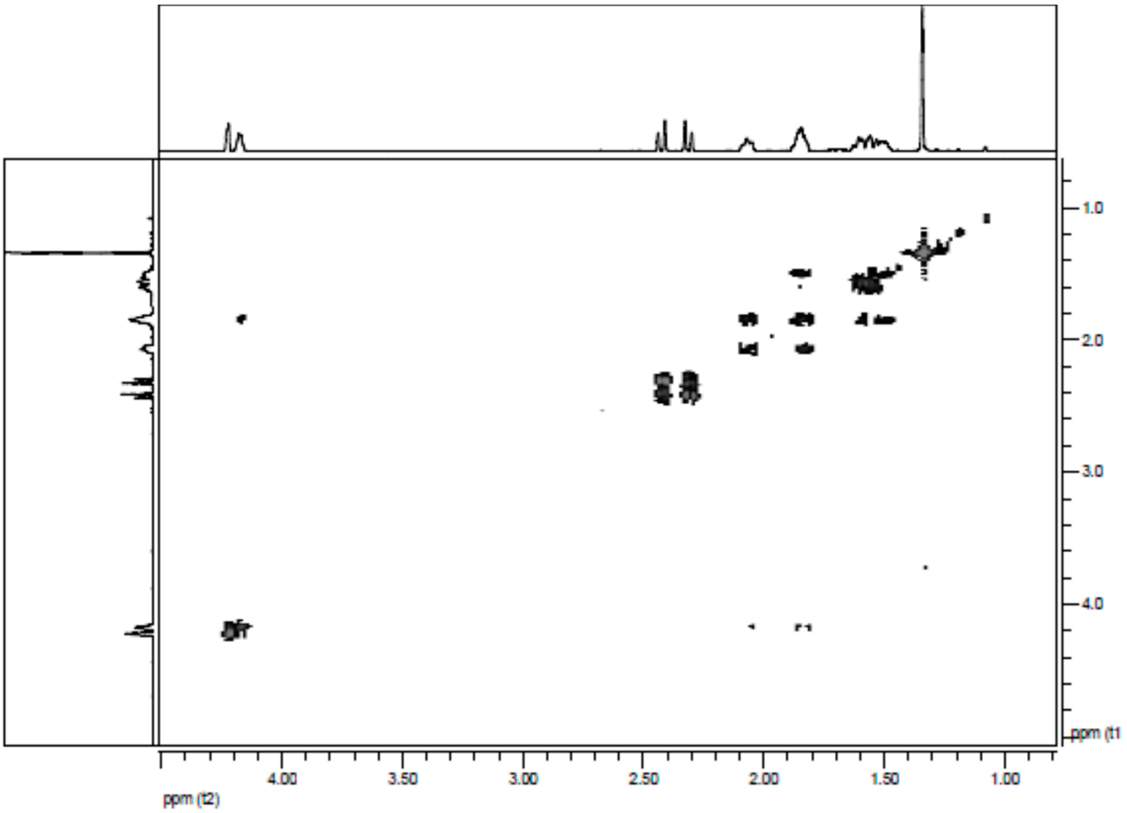

Figure S2. COSY (151 MHz, $CDCl_3$) spectrum of chlorolactone **3**.

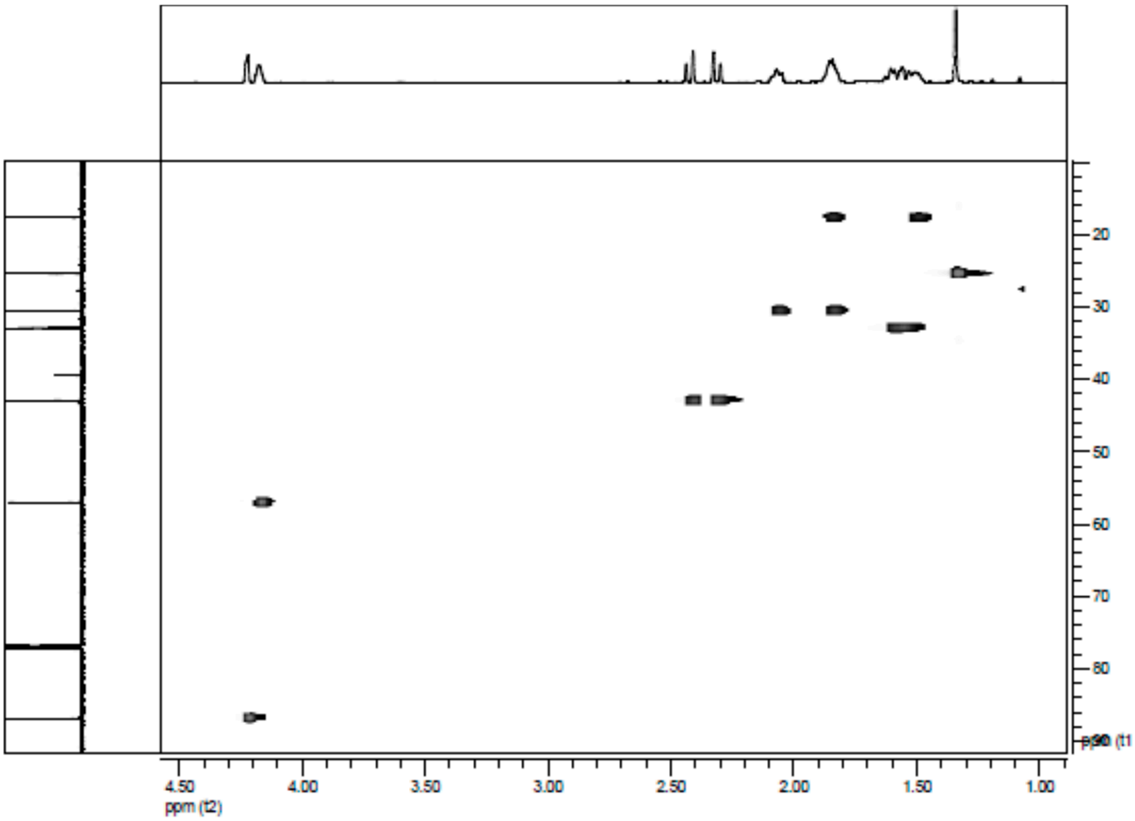

Figure S3. HMQC (151 MHz, $CDCl_3$) spectrum of chlorolactone **3**.

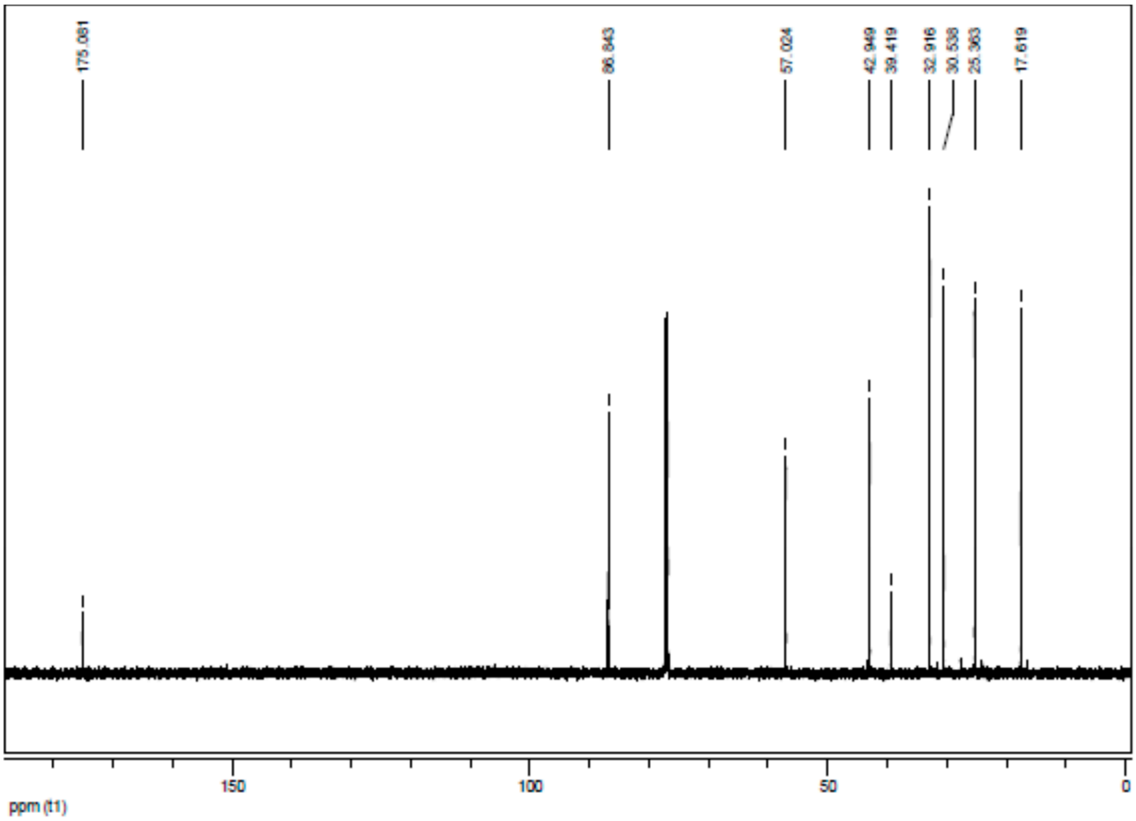

Figure S4. ^{13}C-NMR (151 MHz, $CDCl_3$) spectrum of chlorolactone **3**.

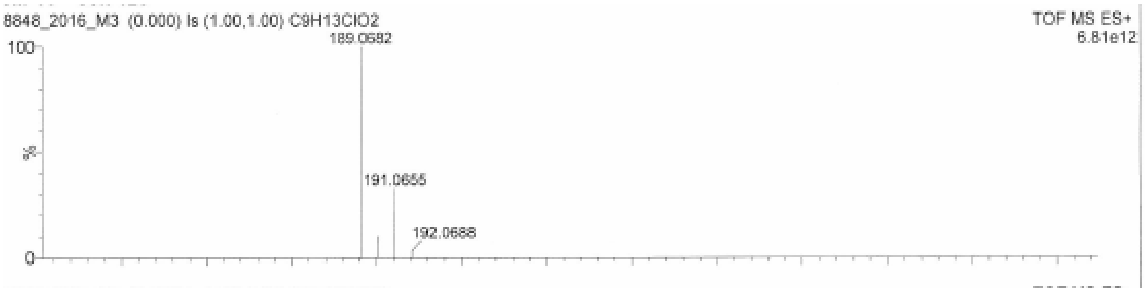

Figure S5. HRMS spectrum of chlorolactone **3**.

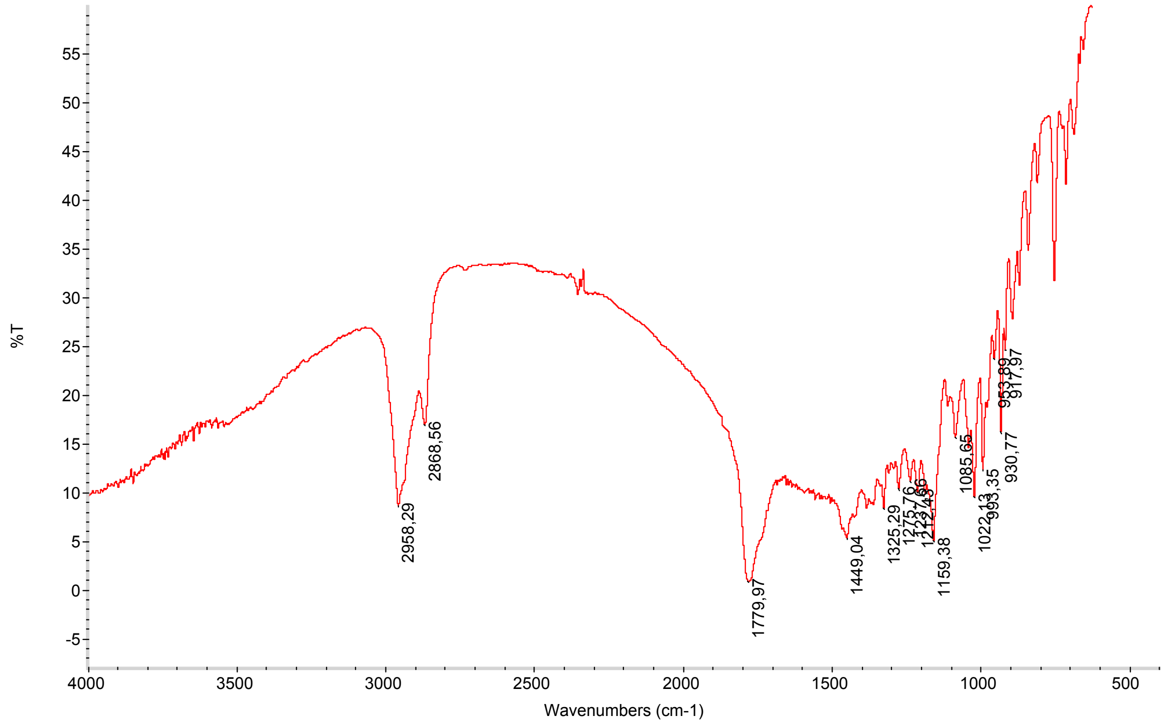

Figure S6. IR spectrum of chlorolactone **3**.

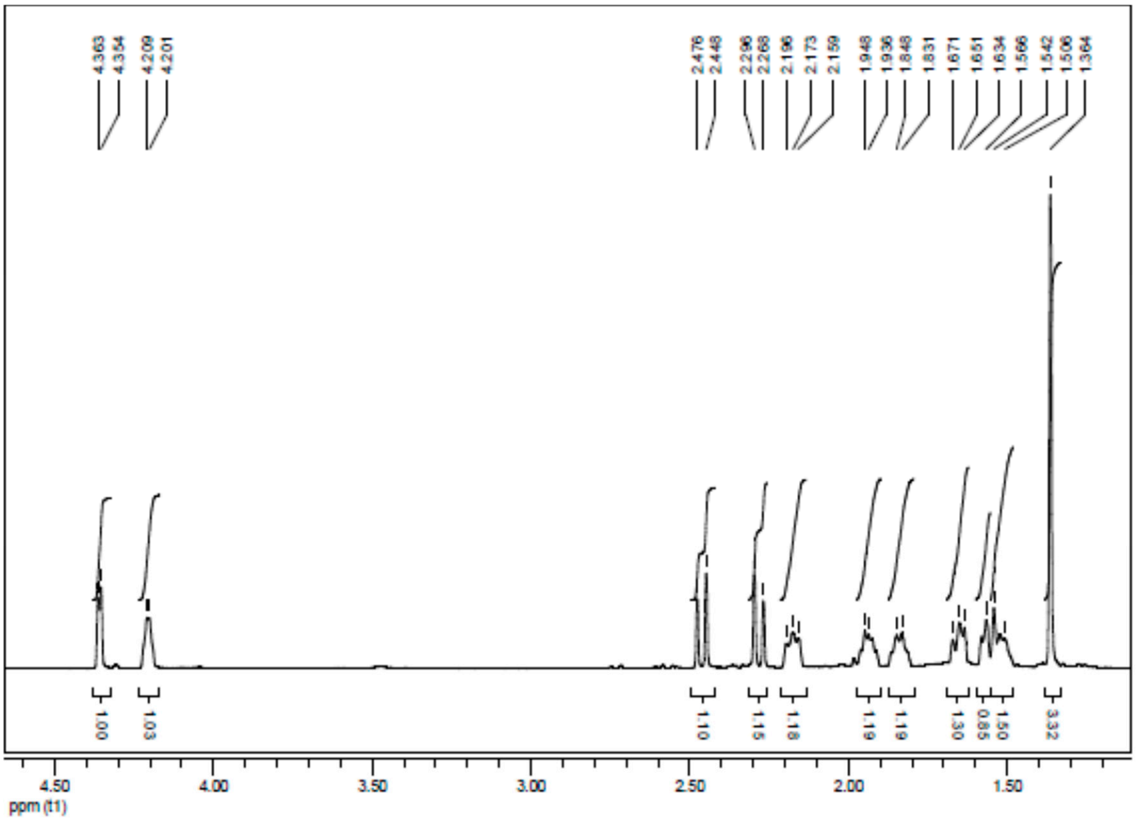

Figure S7. ^{1}H-NMR (600 MHz, $CDCl_3$) spectrum of bromolactone **4**.

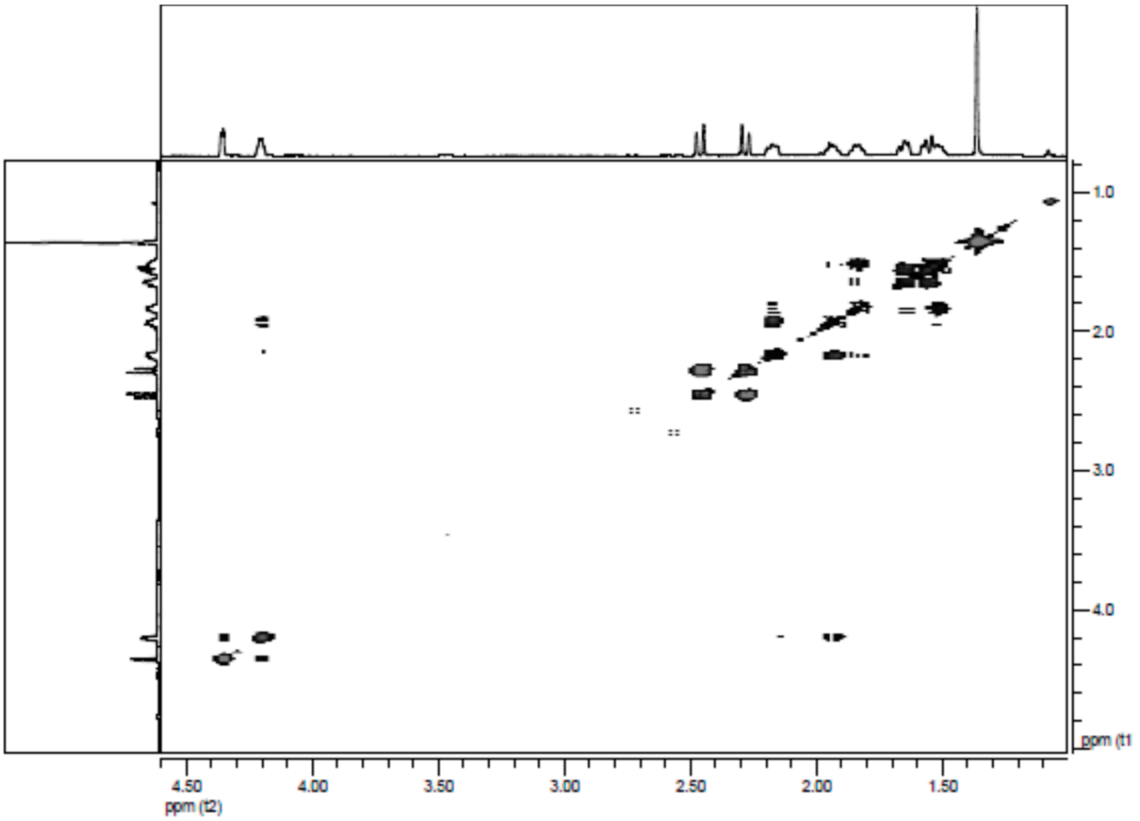

Figure S8. COSY (151 MHz, $CDCl_3$) spectrum of bromolactone **4**.

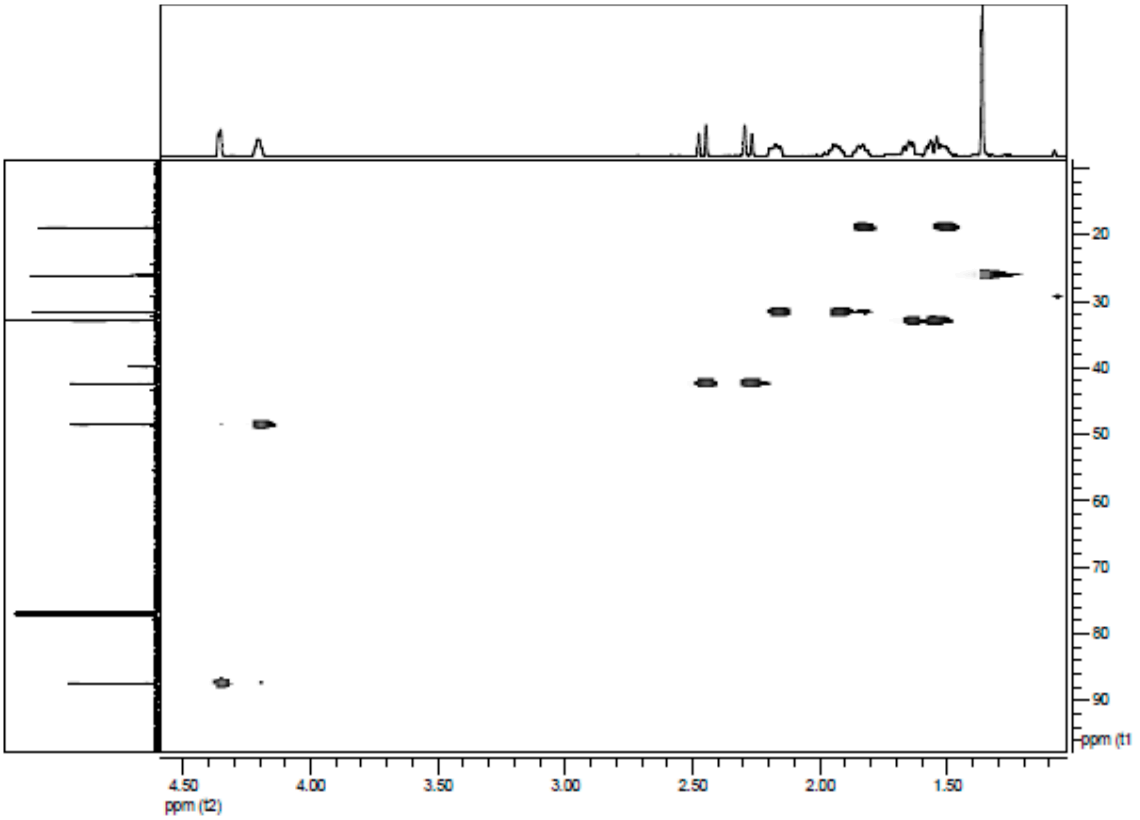

Figure S9. COSY (151 MHz, $CDCl_3$) spectrum of bromolactone **4**.

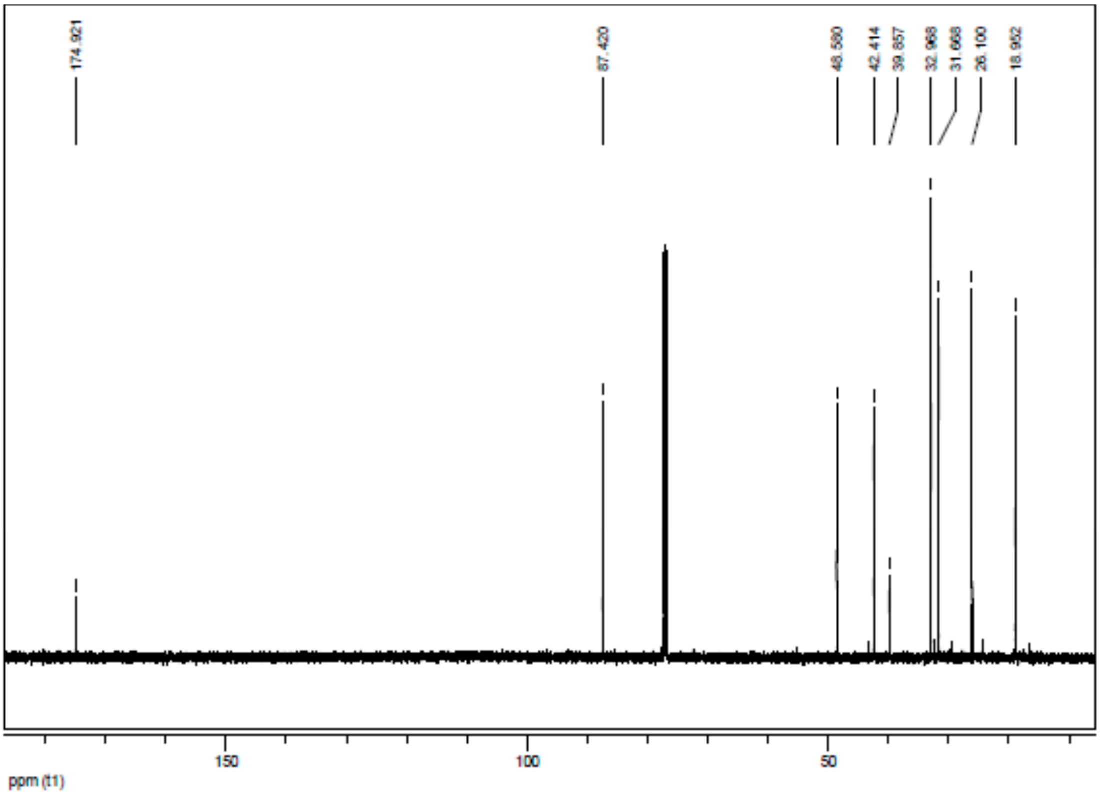

Figure S10. ^{13}C-NMR (151 MHz, $CDCl_3$) spectrum of bromolactone **4**.

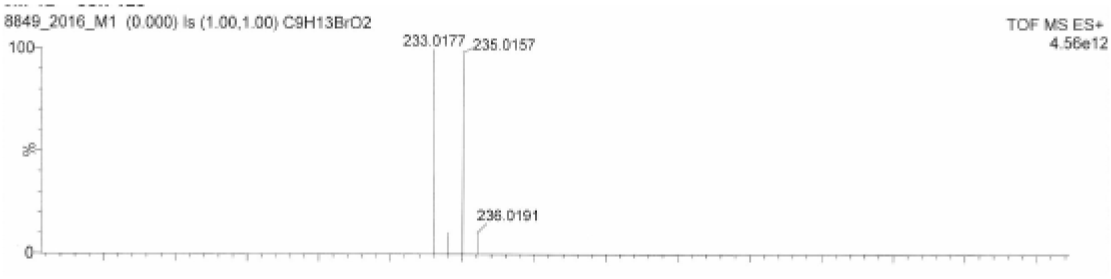

Figure S11. HRMS spectrum of bromolactone **4**.

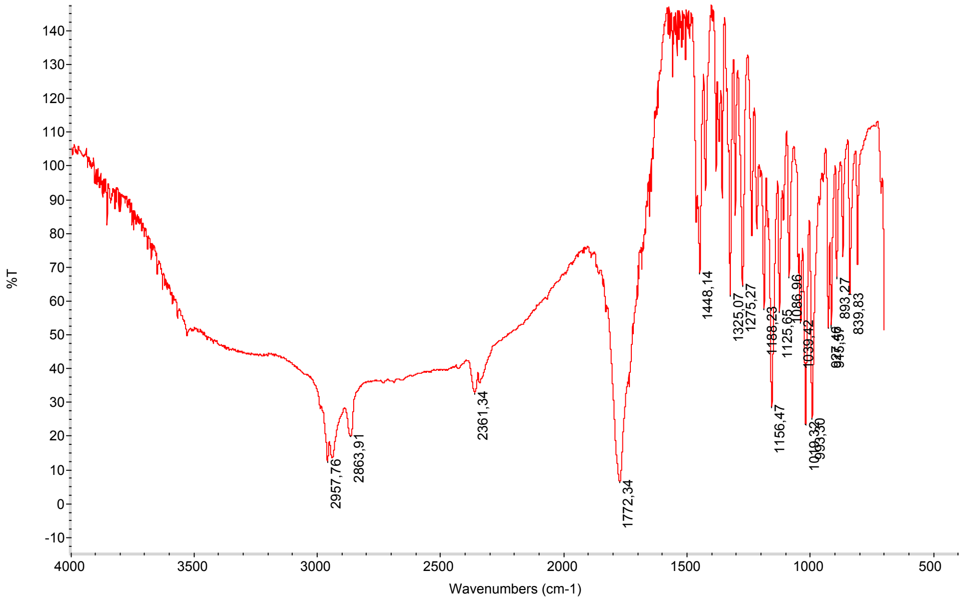

Figure S12. IR spectrum of bromolactone **4**.

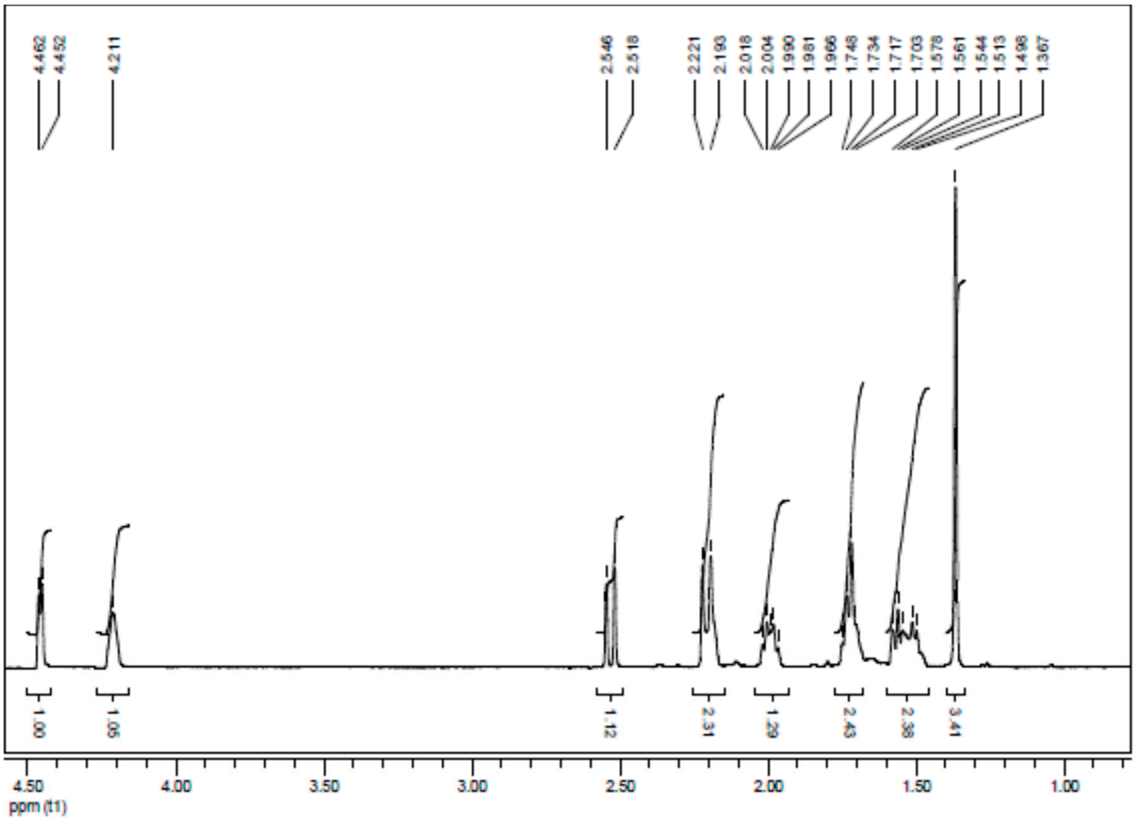

Figure S13. NMR (600 MHz, $CDCl_3$) spectrum of iodolactone **5**.

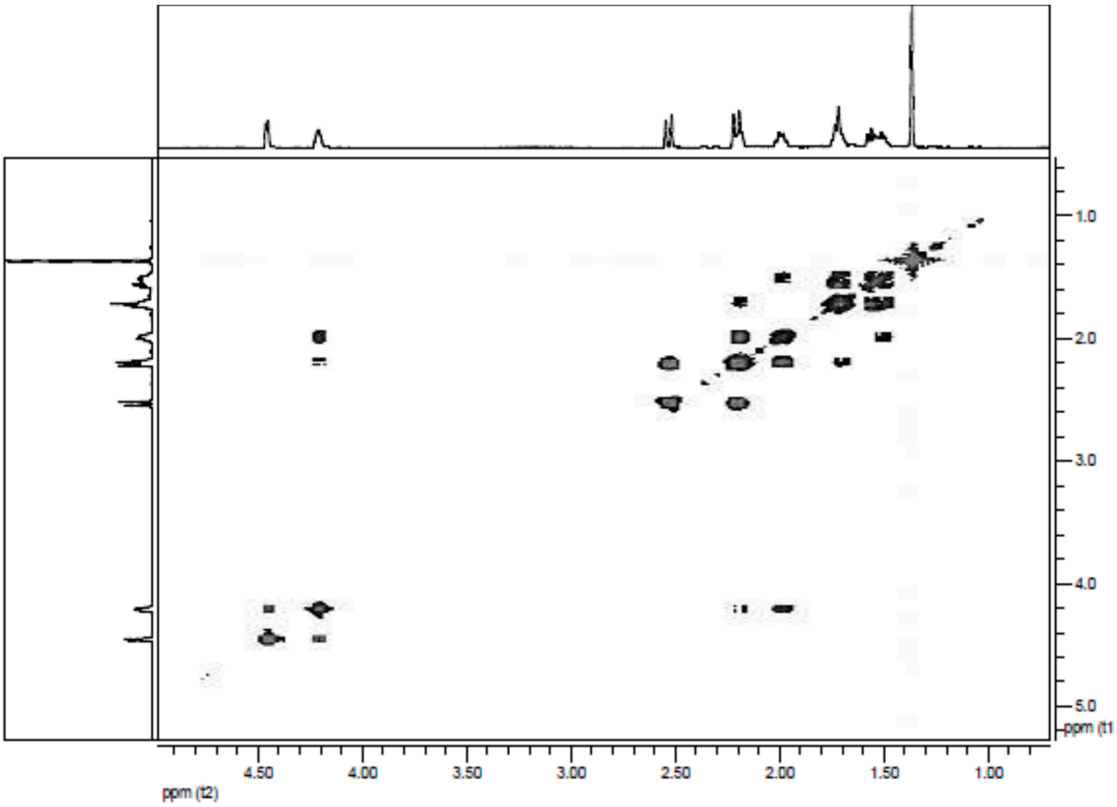

Figure S14. ^{1}H-NMR (600 MHz, $CDCl_3$) spectrum of iodolactone **5**.

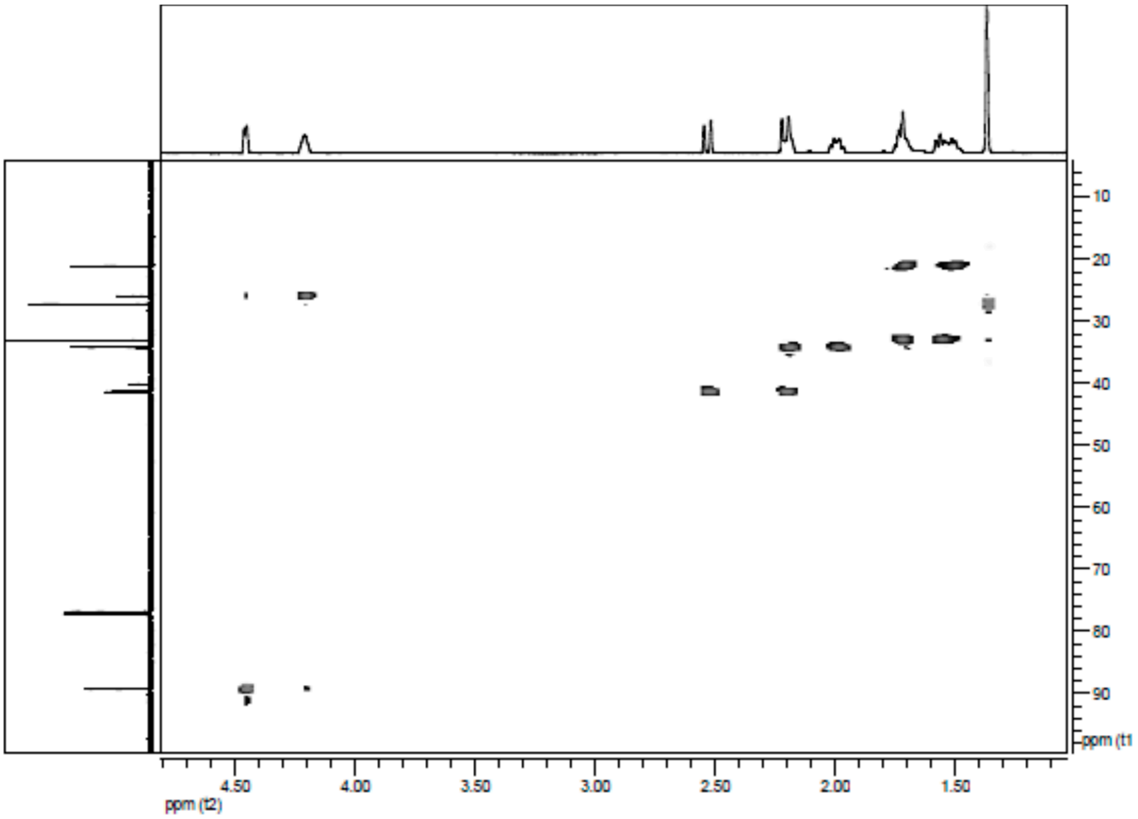

Figure S15. ^{13}C-NMR (151 MHz, $CDCl_3$) spectrum of iodolactone **5**.

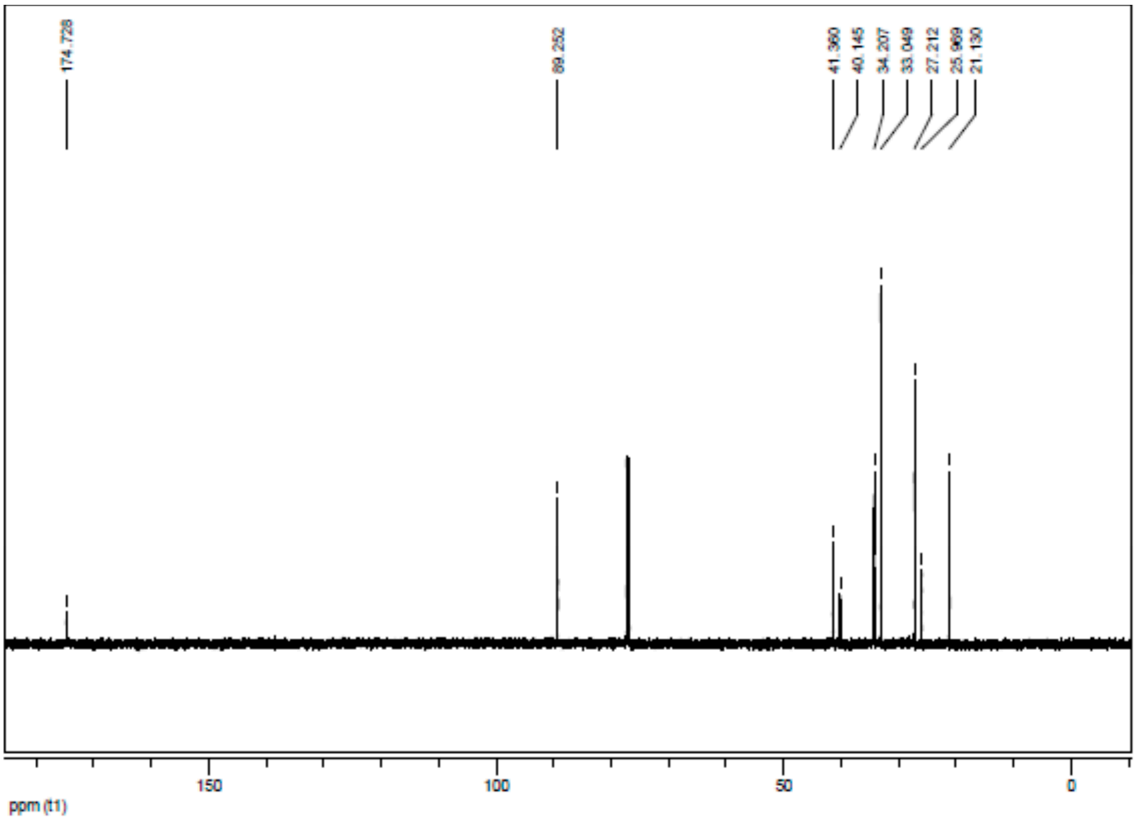

Figure S16. ^{13}C-NMR (151 MHz, $CDCl_3$) spectrum of iodolactone **5**.

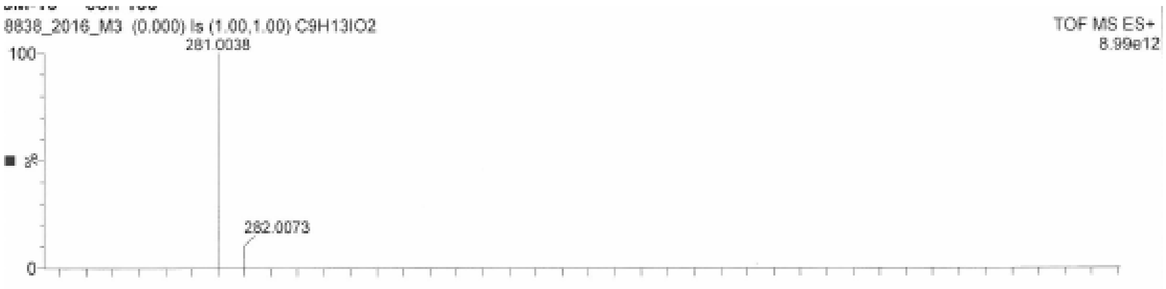

Figure S17. HRMS spectrum of iodolactone **5**.

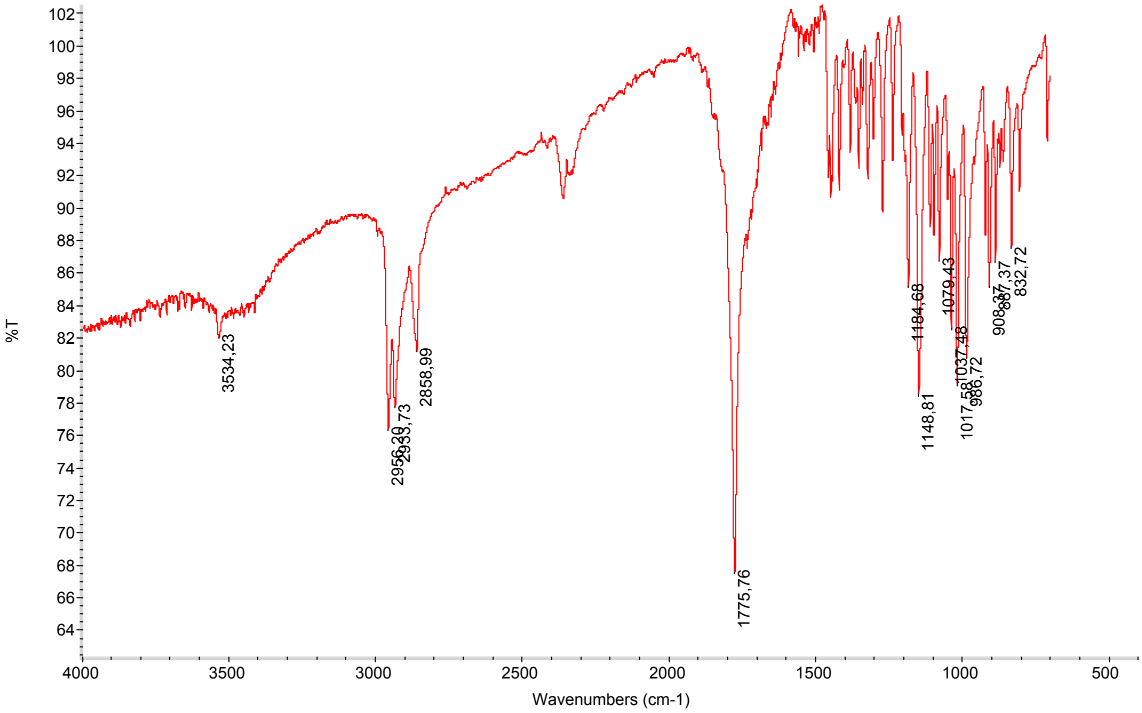

Figure S18. IR spectrum of iodolactone **5**.

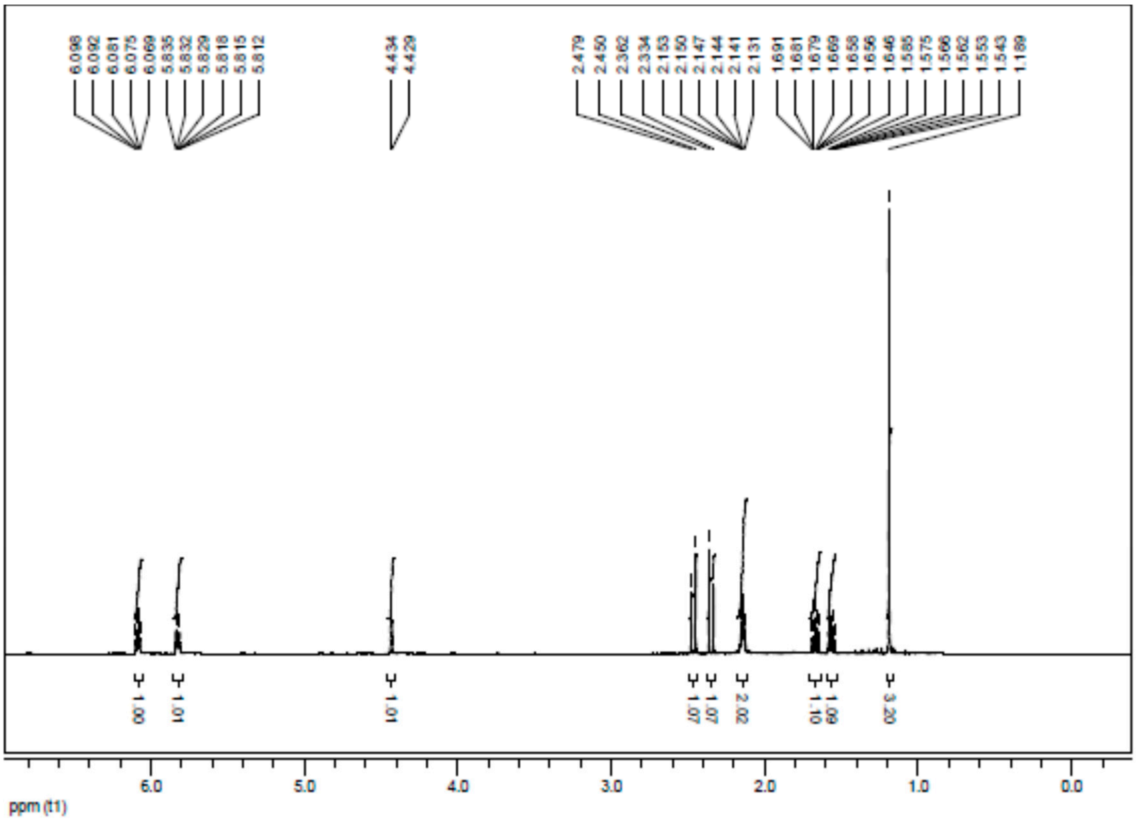

Figure S19. ^{1}H-NMR (600 MHz, $CDCl_3$) spectrum of unsaturated lactone **6**.

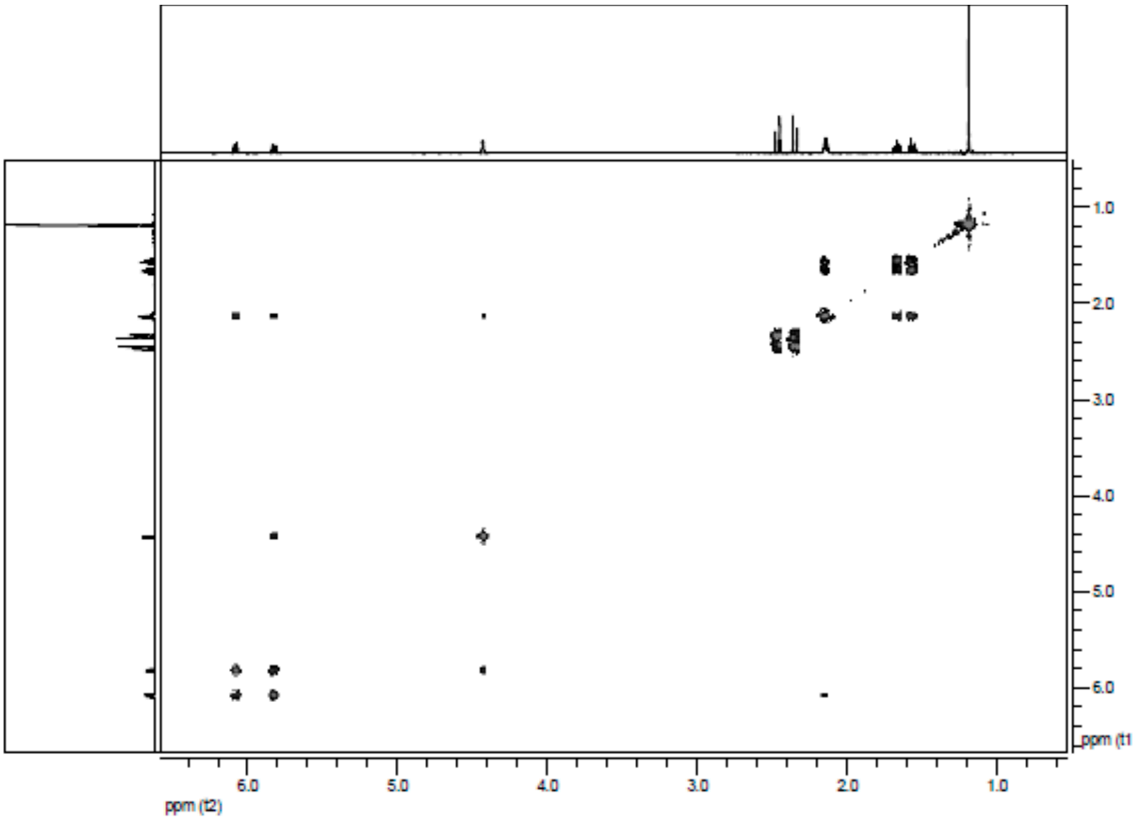

Figure S20. COSY (151 MHz, $CDCl_3$) spectrum of unsaturated lactone **6**.

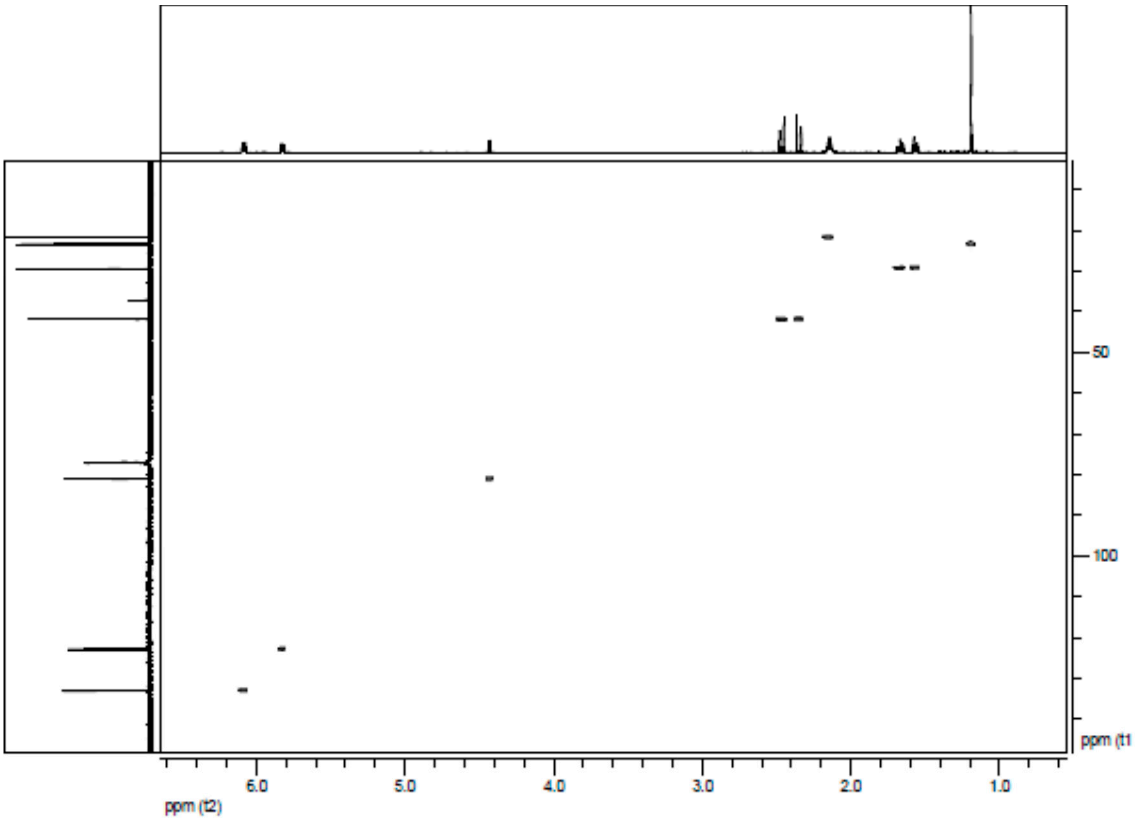

Figure S21. HMQC (151 MHz, $CDCl_3$) spectrum of unsaturated lactone **6**.

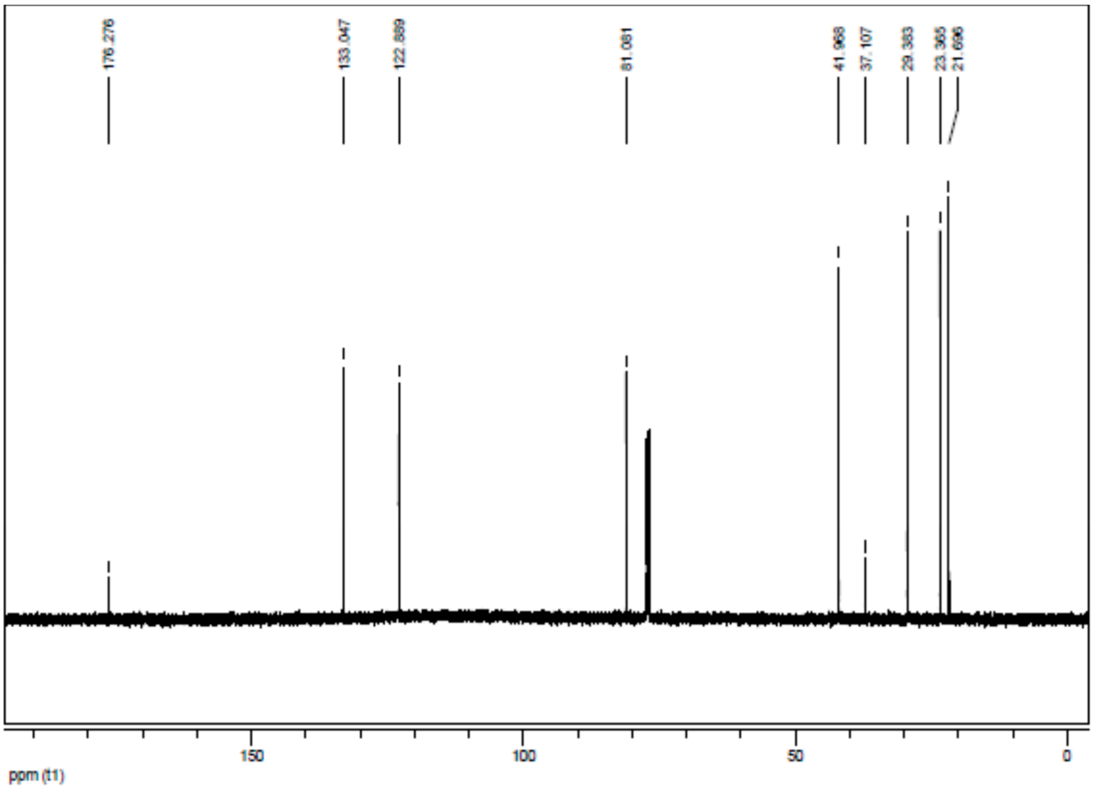

Figure S22. ^{13}C-NMR (151 MHz, $CDCl_3$) spectrum of unsaturated lactone **6**.

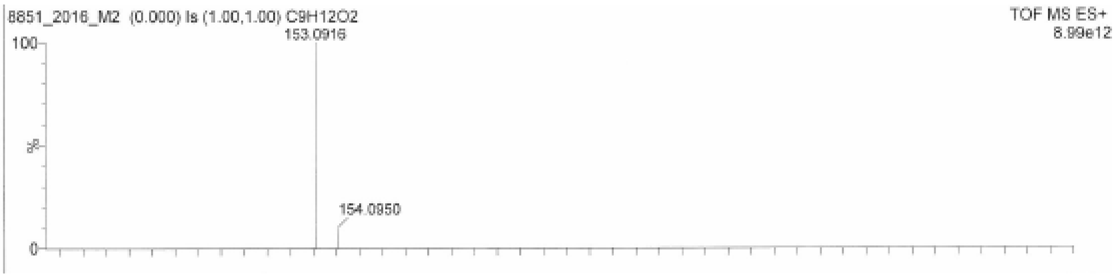

Figure S23. HRMS spectrum of unsaturated lactone **6**.

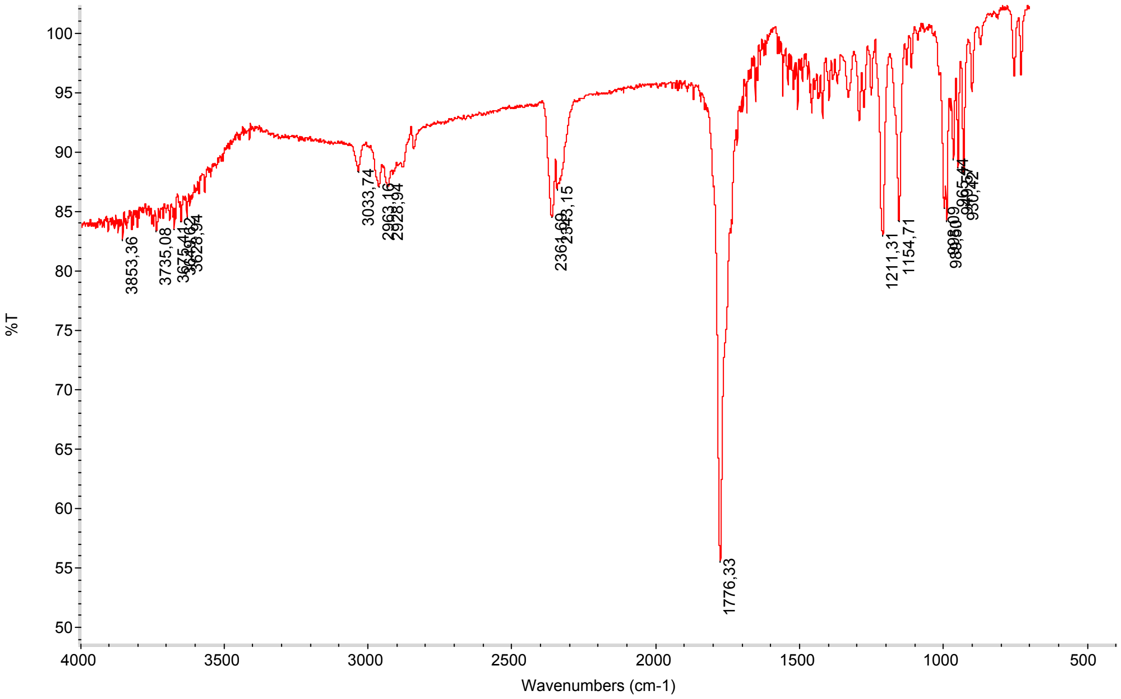

Figure S24. IR spectrum of unsaturated lactone **6**.

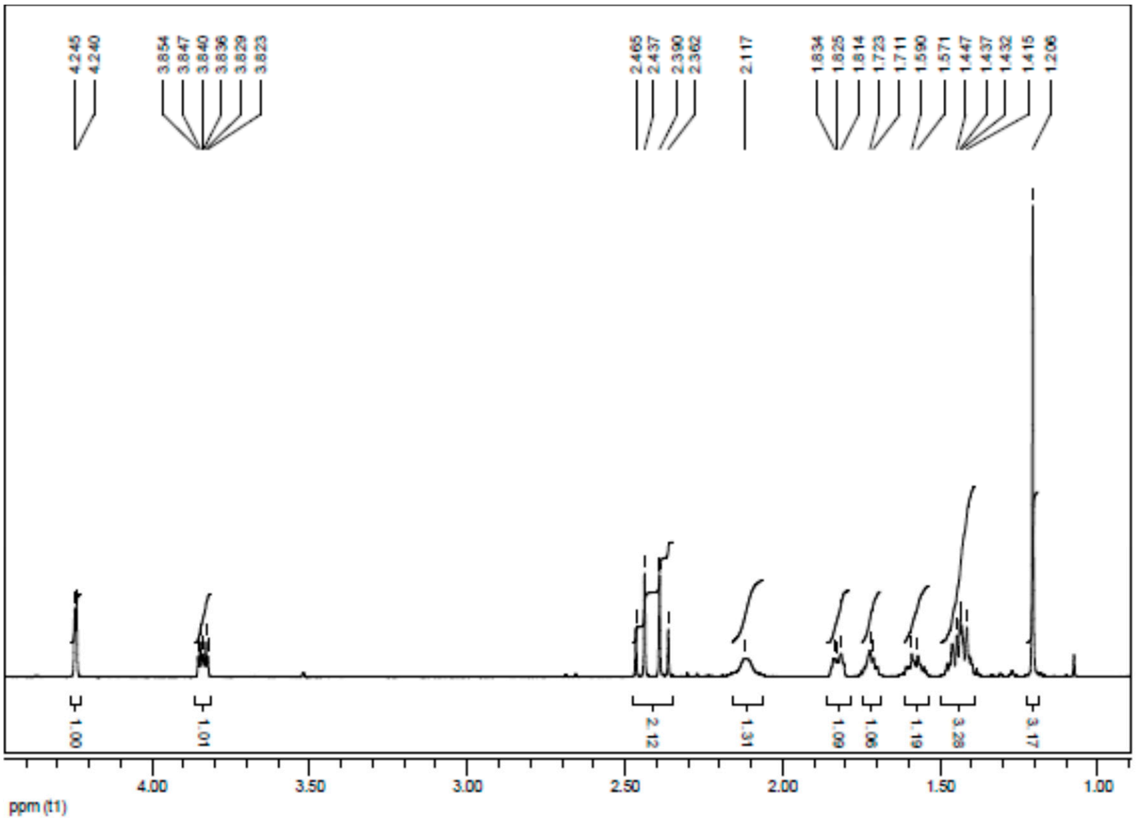

Figure S25. ^{1}H-NMR (600 MHz, $CDCl_3$) spectrum of hydroxylactone **7**.

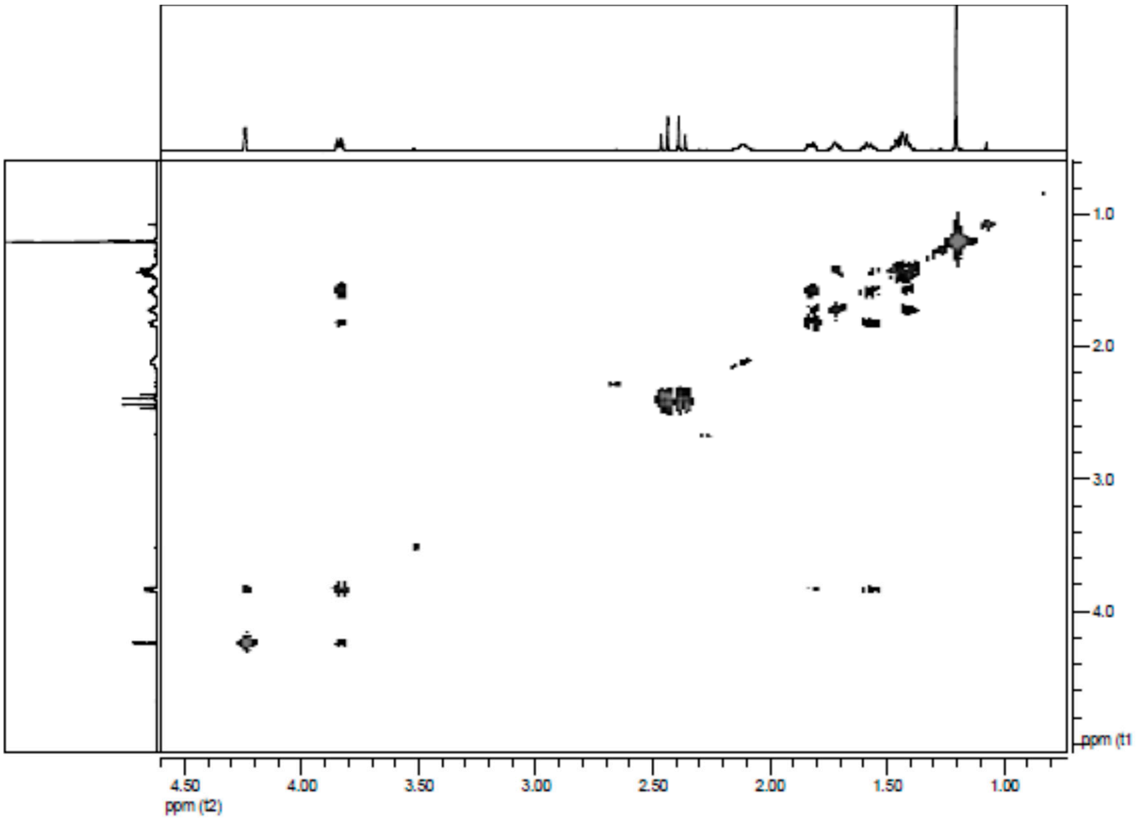

Figure S26. COSY (151 MHz, $CDCl_3$) spectrum of hydroxylactone **7**.

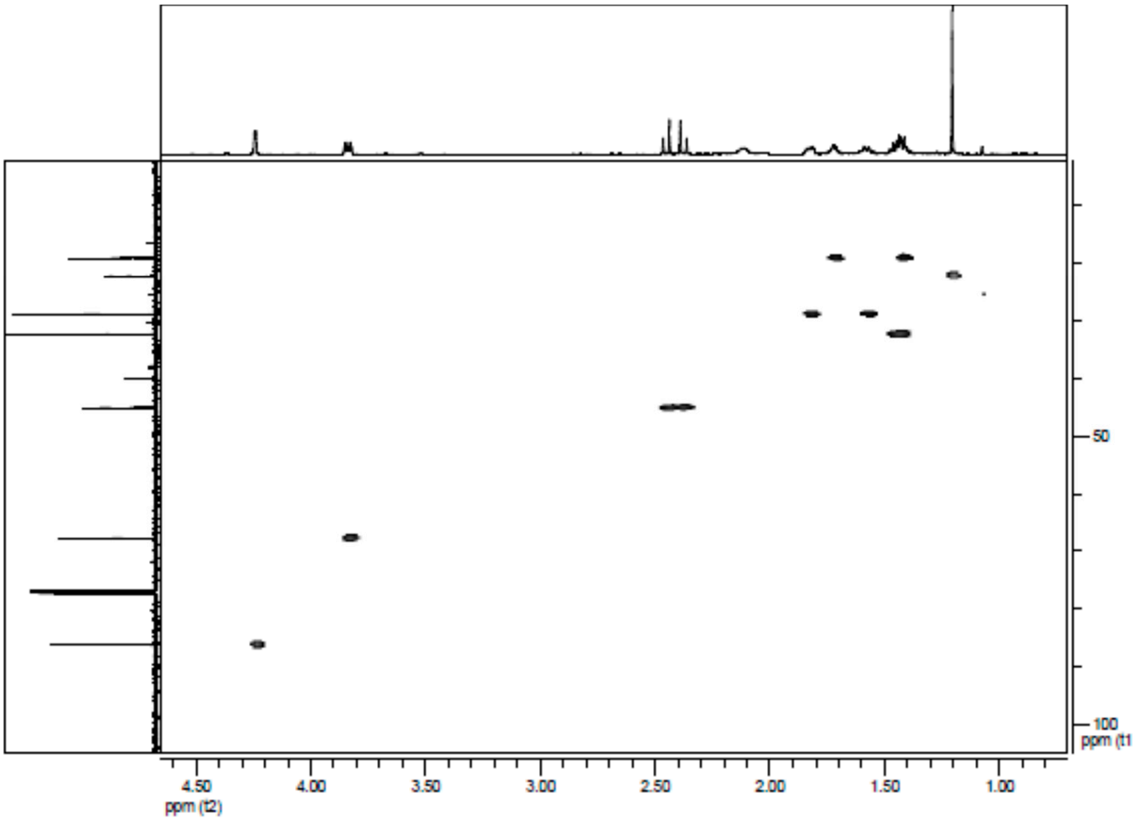

Figure S27. HMQC (151 MHz, $CDCl_3$) spectrum of hydroxylactone **7**.

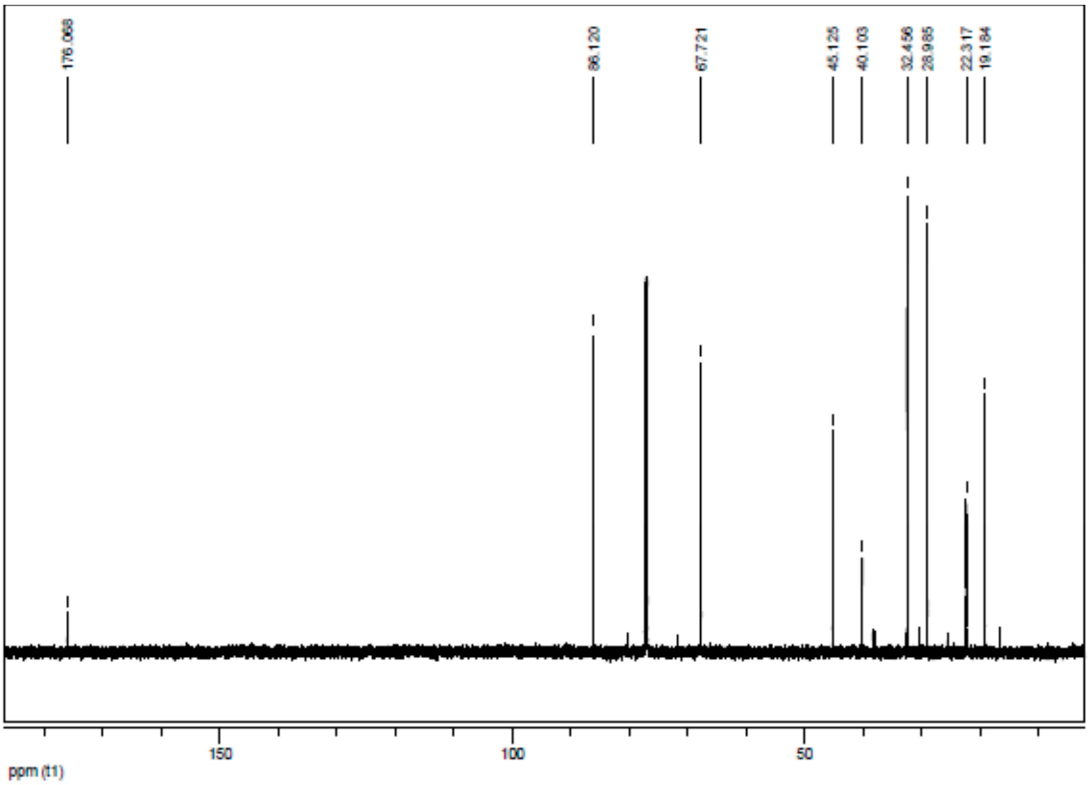

Figure S28. ^{13}C-NMR (151 MHz, $CDCl_3$) spectrum of hydroxylactone **7**.

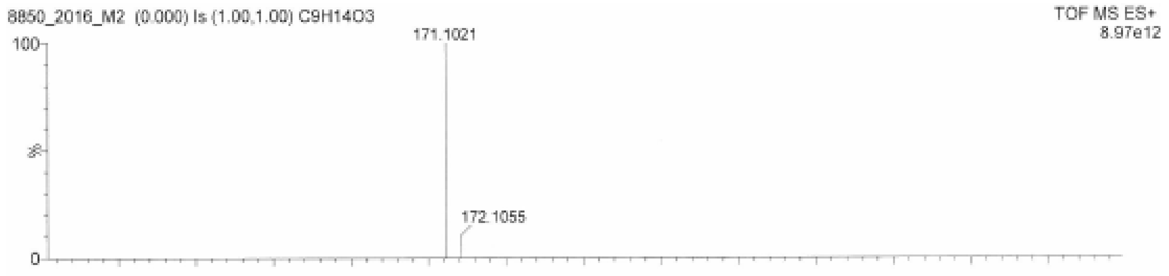

Figure S29. HRMS spectrum of hydroxylactone **7**.

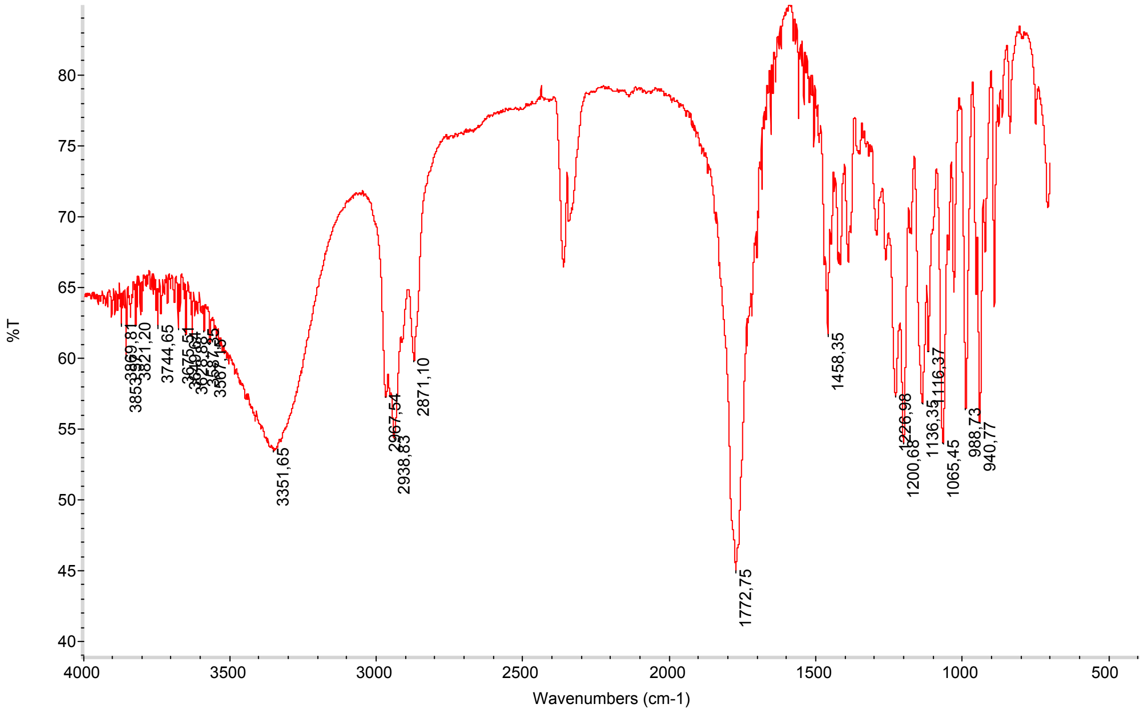

Figure S30. IR spectrum of hydroxylactone **7**.

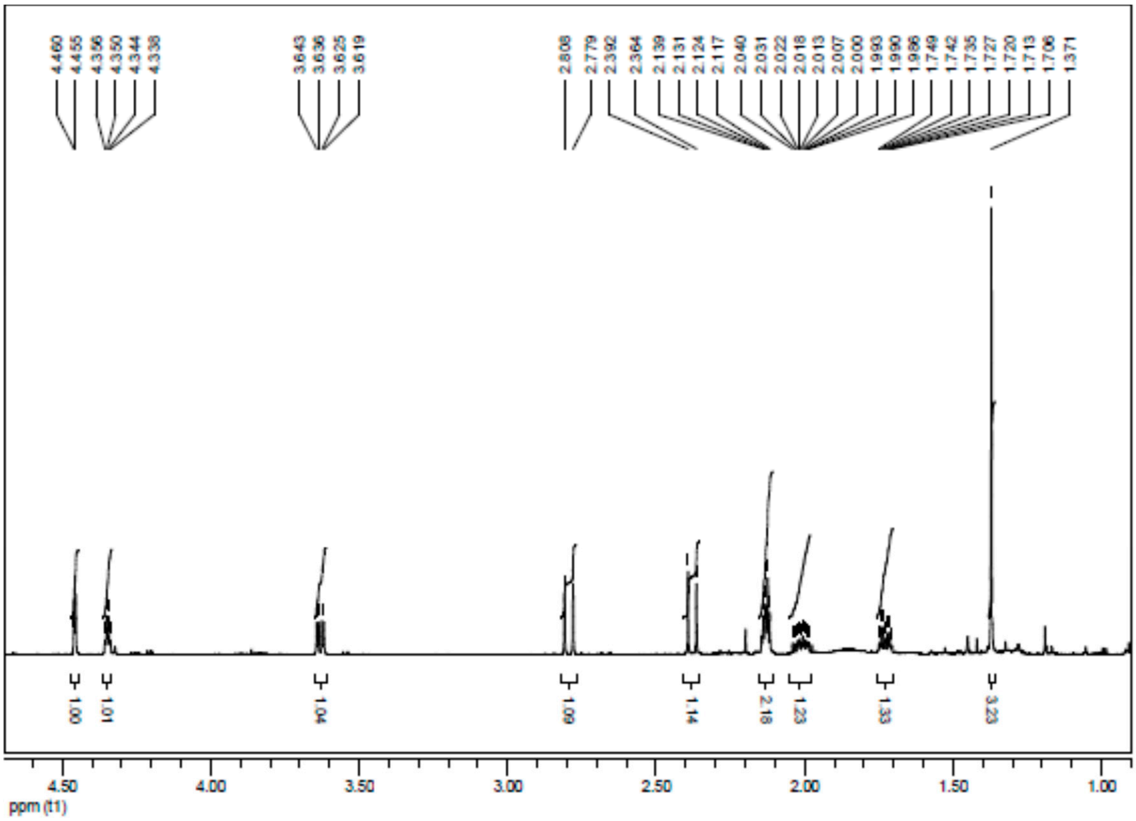

Figure S31. ¹H-NMR (600 MHz, $CDCl_3$) spectrum of hydroxy-chlorolactone **8**.

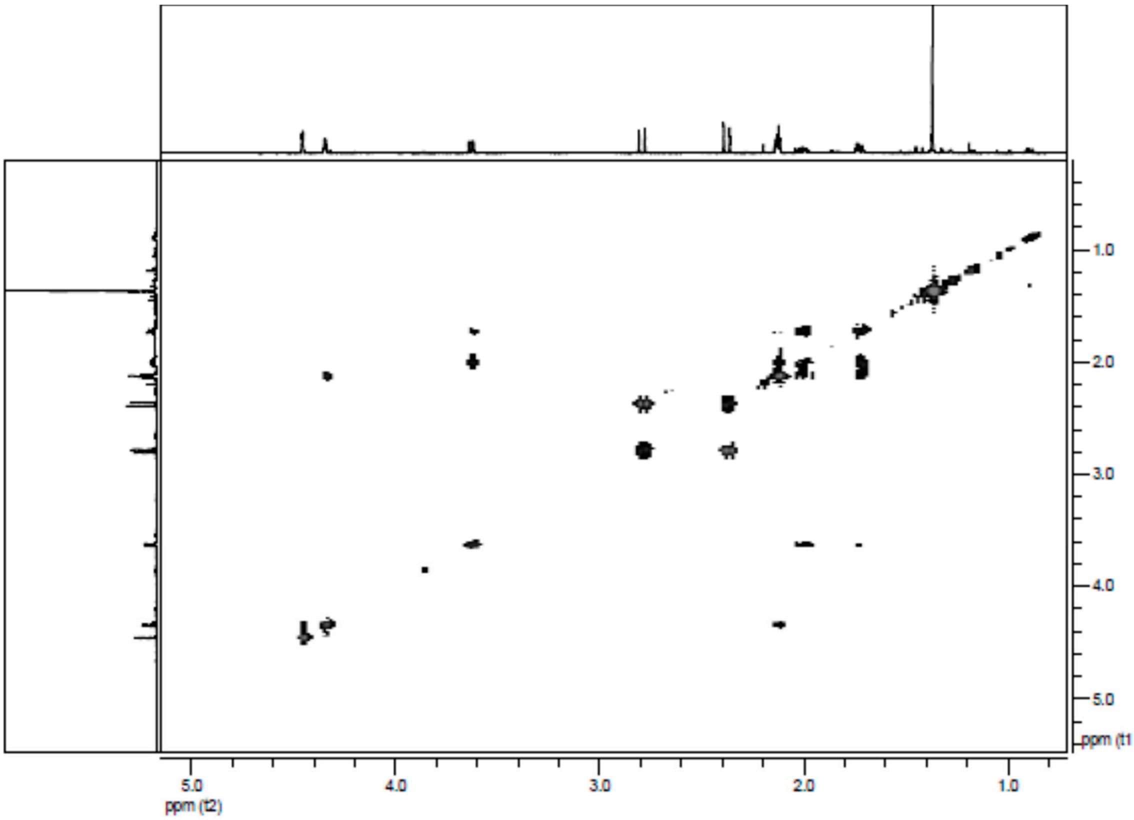

Figure 32. COSY (151 MHz, $CDCl_3$) spectrum of hydroxy-chlorolactone **8**.

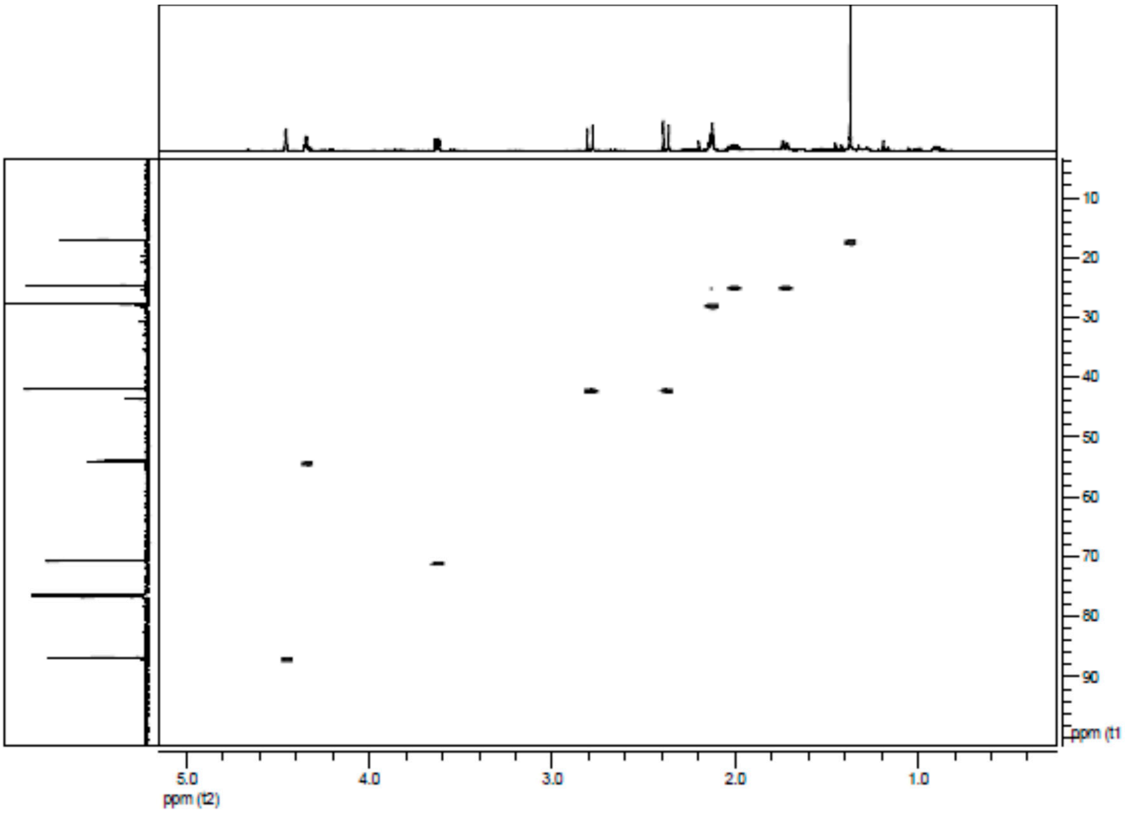

Figure S33. HMQC (151 MHz, $CDCl_3$) spectrum of hydroxy-chlorolactone **8**.

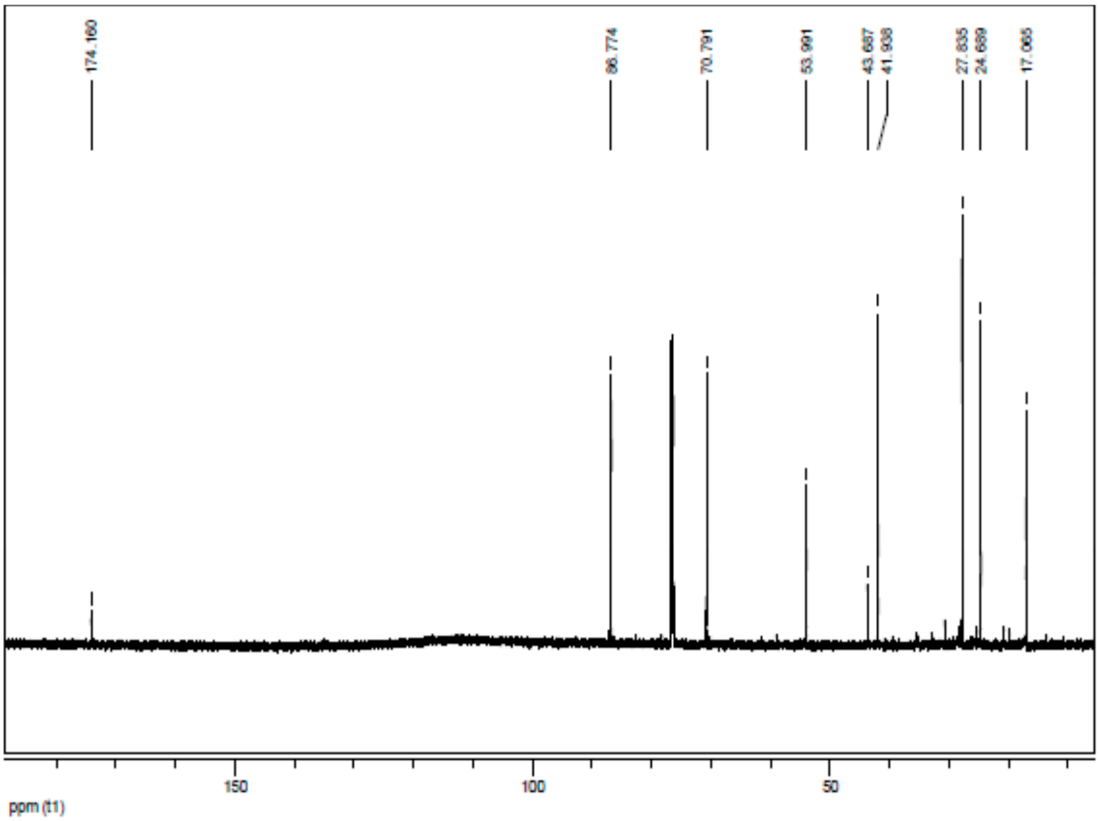

Figure S34. ^{13}C-NMR (151 MHz, $CDCl_3$) spectrum of hydroxy-chlorolactone **8**.

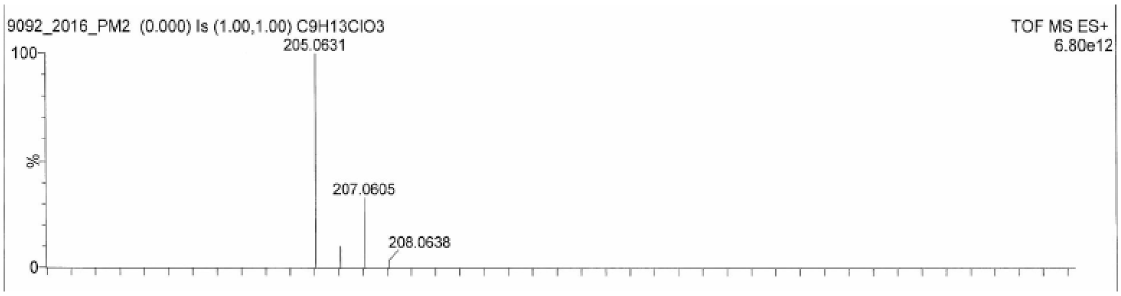

Figure S35. HRMS spectrum of hydroxy-chlorolactone **8**.

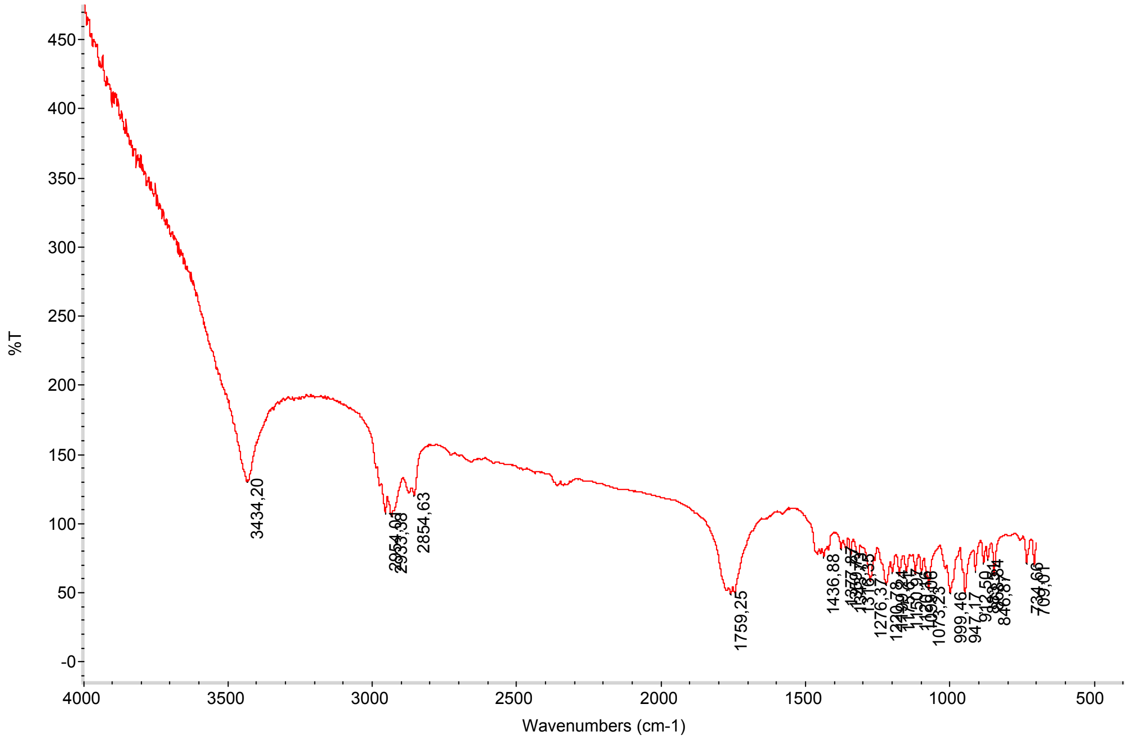

Figure S36. IR spectrum of hydroxy-chlorolactone **8**.

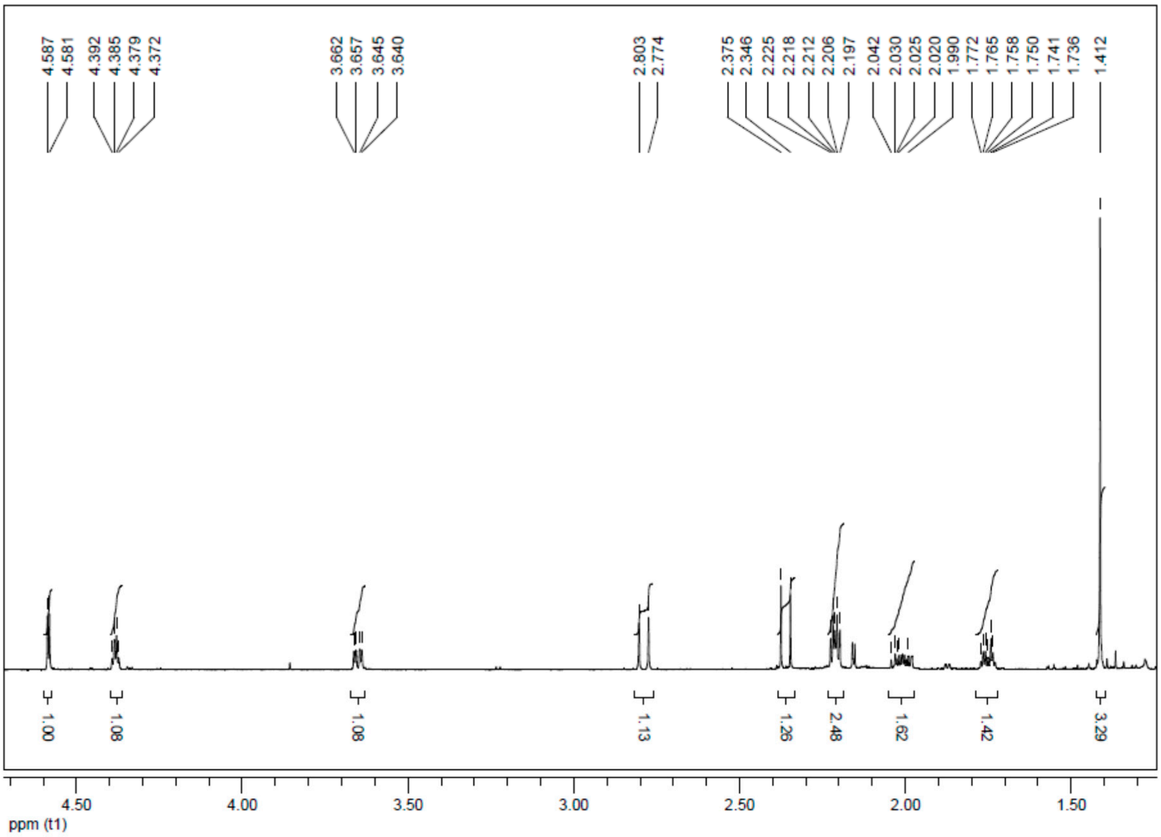

Figure S37. ^{1}H-NMR (600 MHz, $CDCl_3$) spectrum of hydroxy-bromolactone **9**.

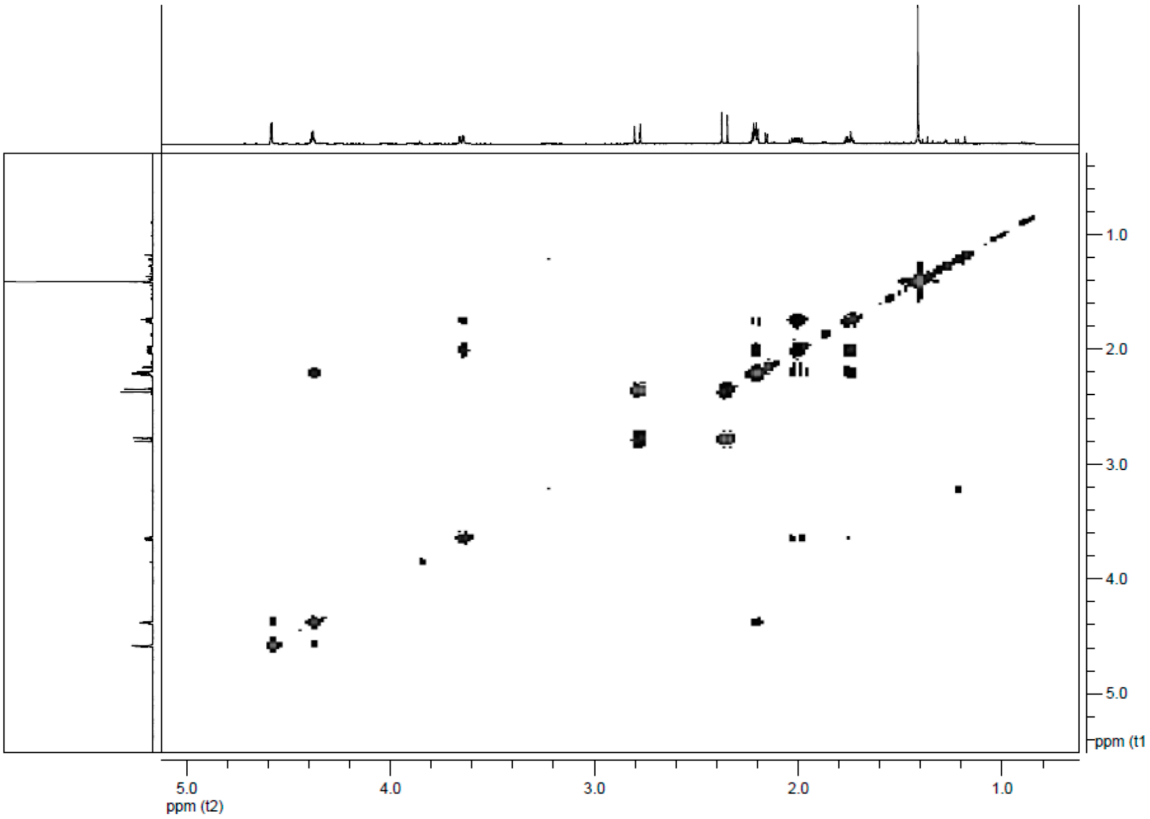

Figure S38. COSY (151 MHz, $CDCl_3$) spectrum of hydroxy-bromolactone **9**.

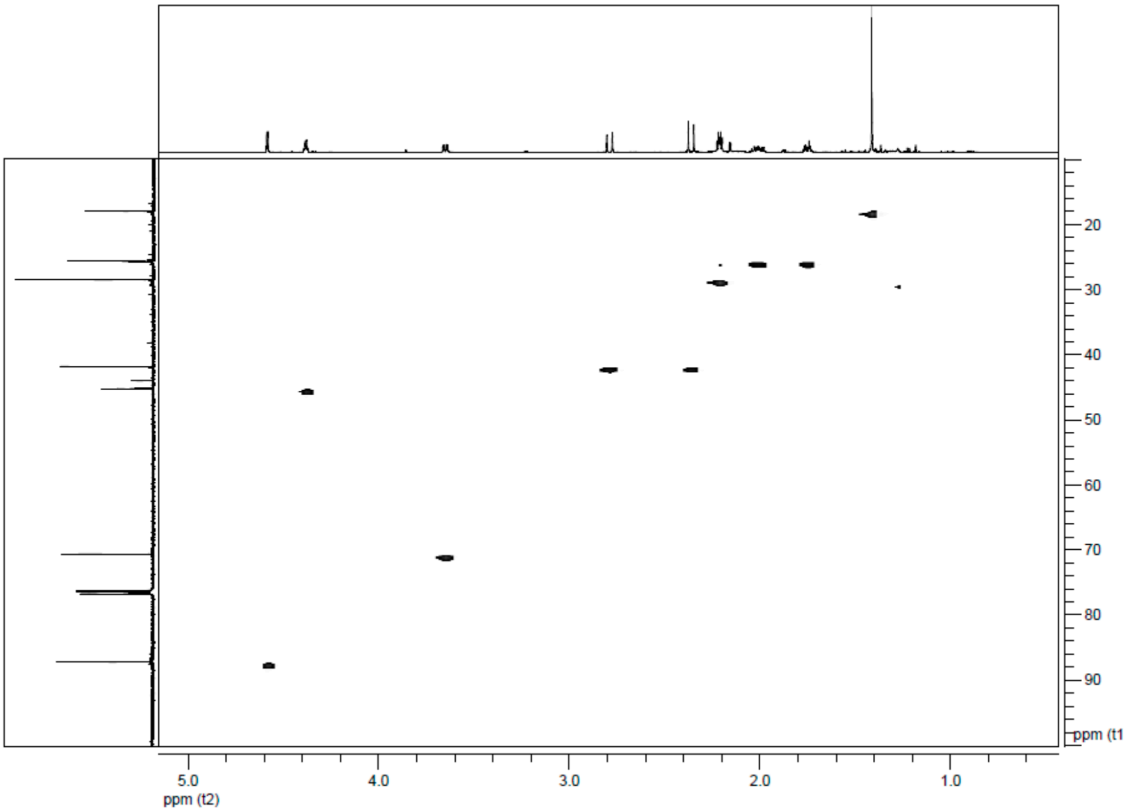

Figure S39. HMQC (151 MHz, $CDCl_3$) spectrum of hydroxy-bromolactone **9**.

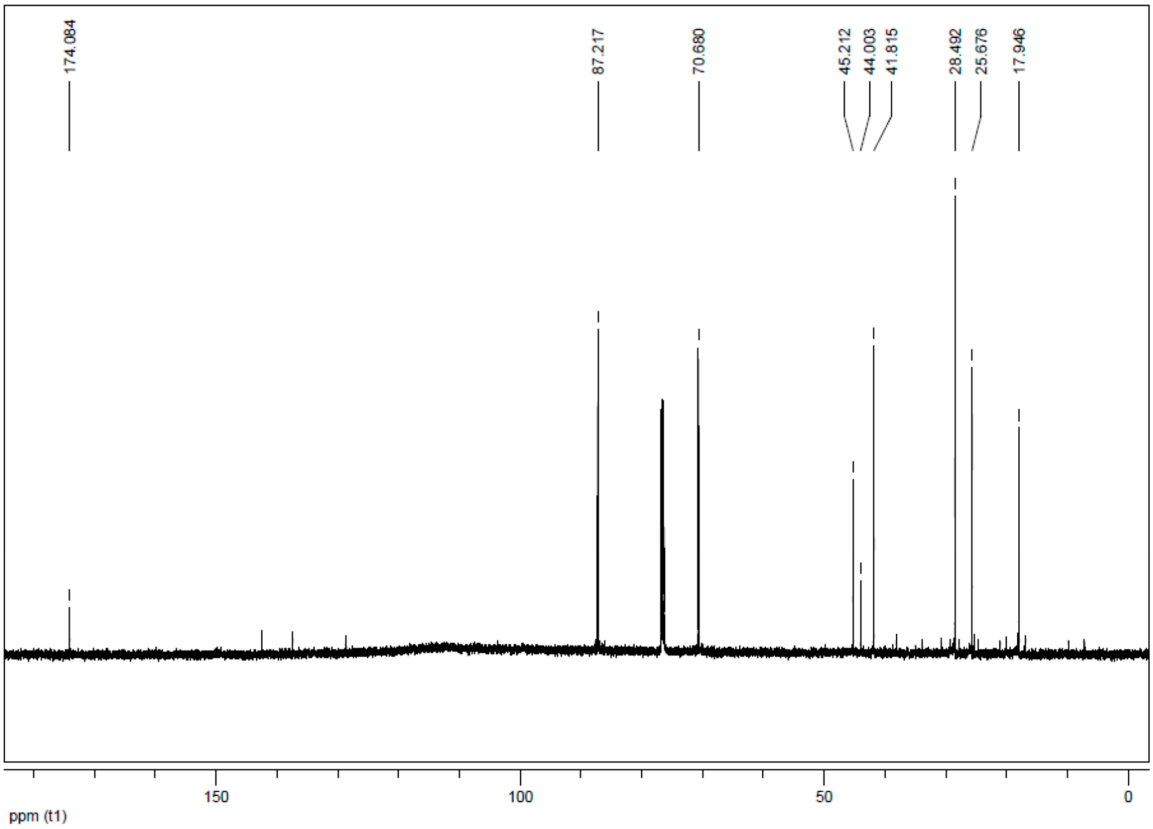

Figure S40. ^{13}C-NMR (151 MHz, $CDCl_3$) spectrum of hydroxy-bromolactone **9**.

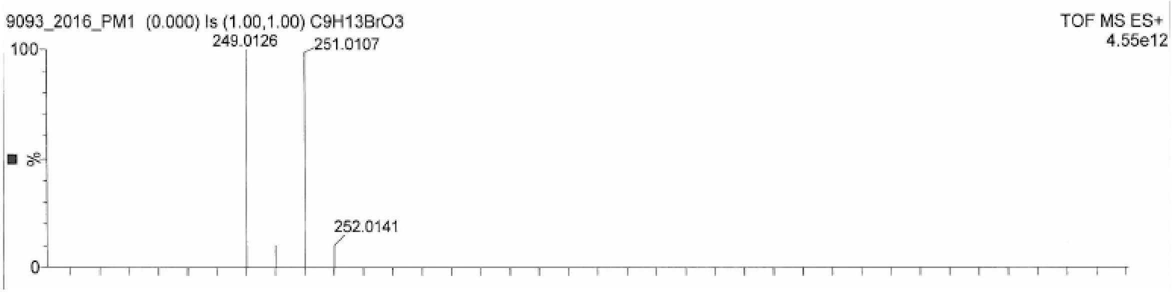

Figure S41. HRMS spectrum of hydroxy-bromolactone **9**.

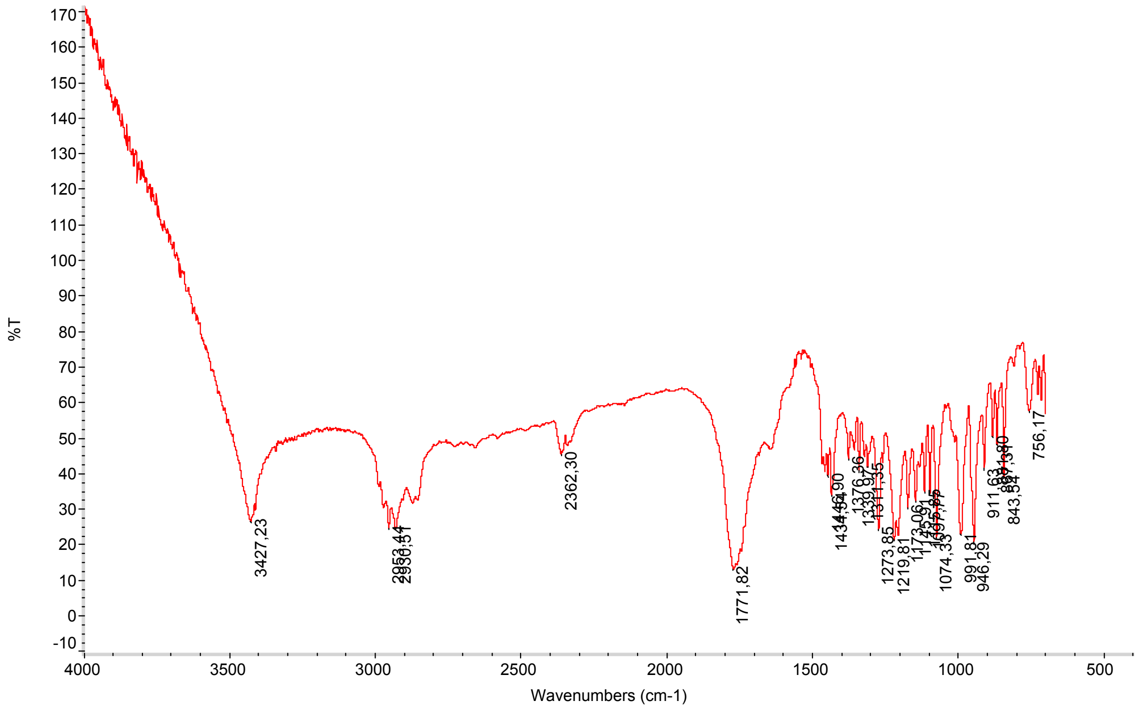

Figure S42. IR spectrum of hydroxy-bromolactone **9**.

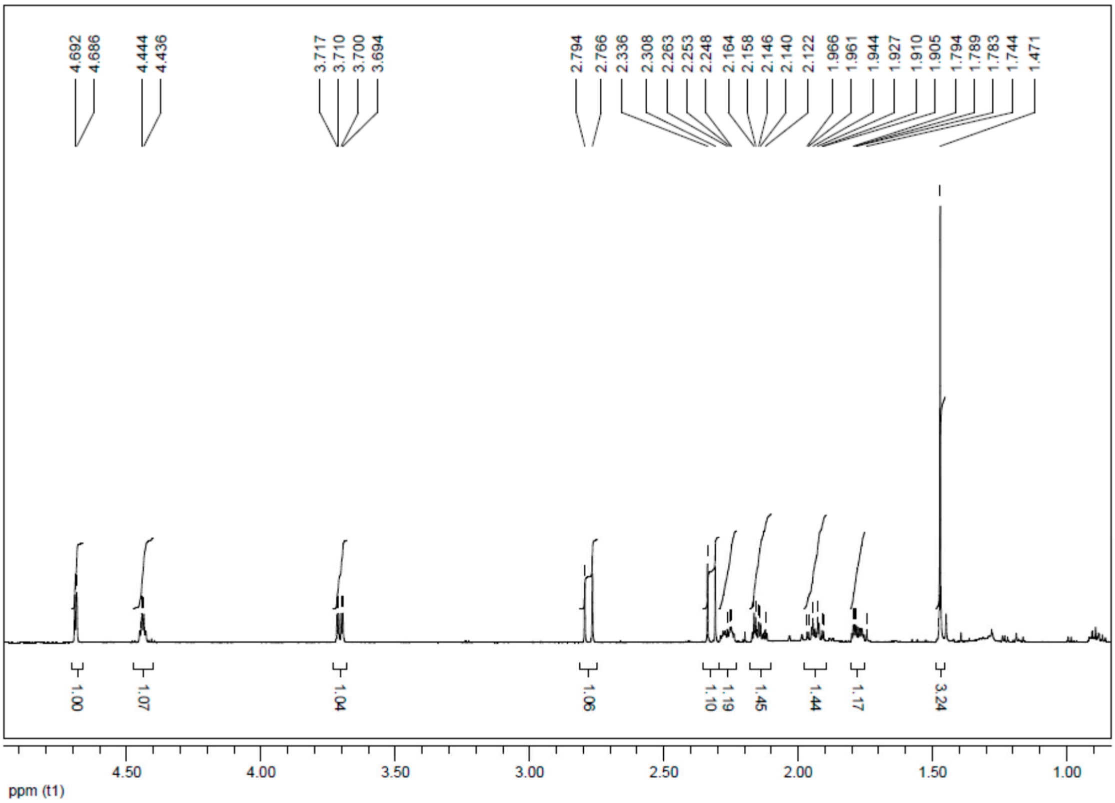

Figure S43. ^{1}H-NMR (600 MHz, $CDCl_3$) spectrum of hydroxy-iodolactone **10**.

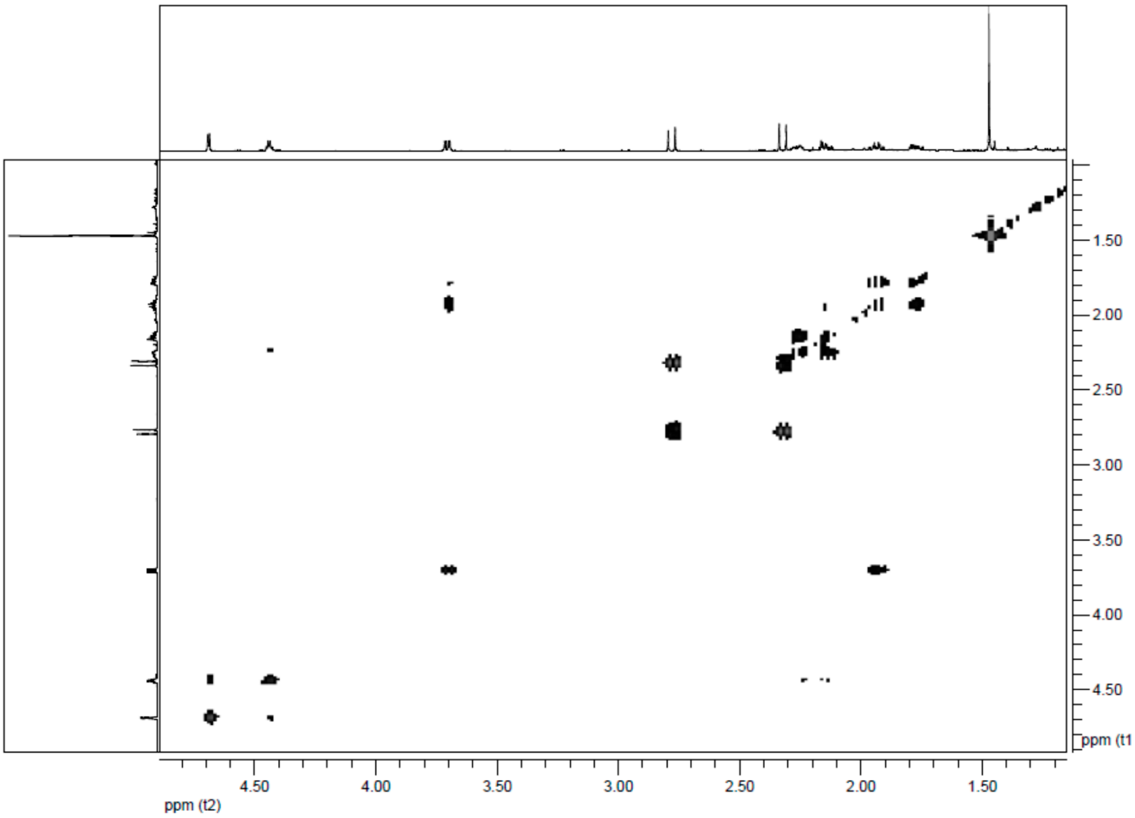

Figure S44. COSY (151 MHz, $CDCl_3$) spectrum of hydroxy-iodolactone **10**.

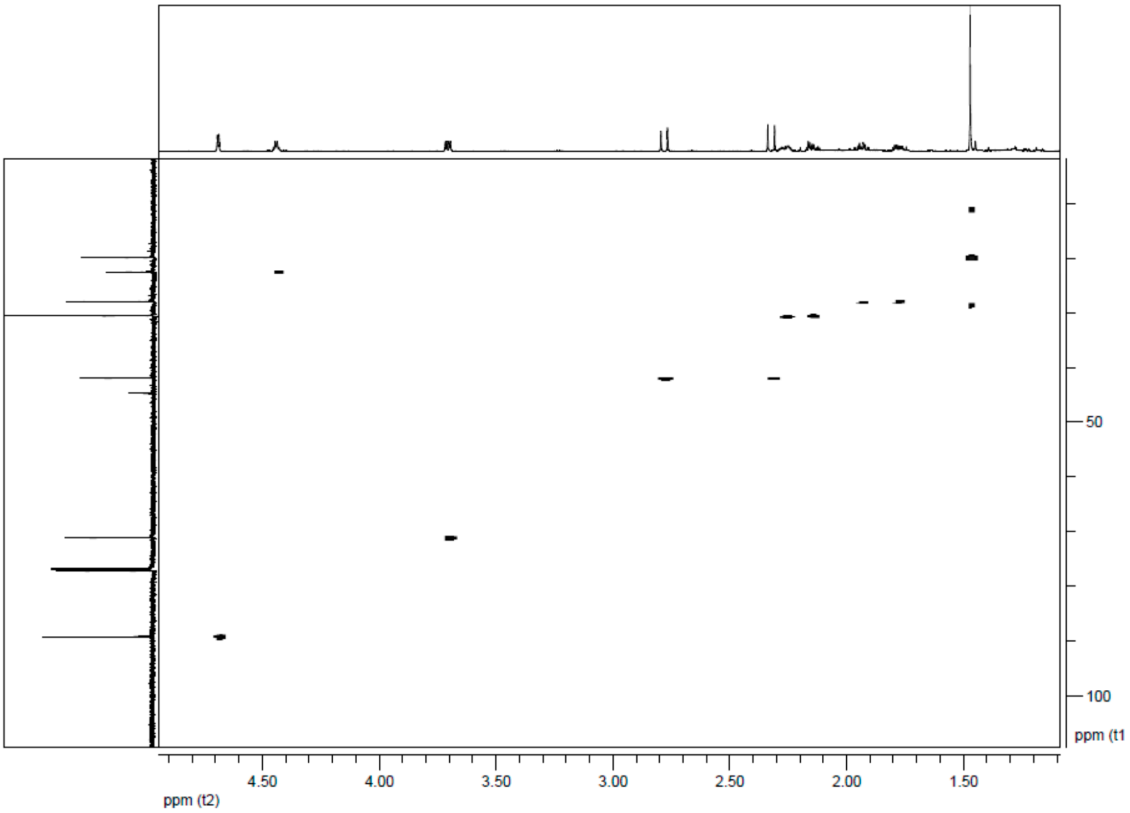

Figure S45. HMQC (151 MHz, $CDCl_3$) spectrum of hydroxy-iodolactone **10**.

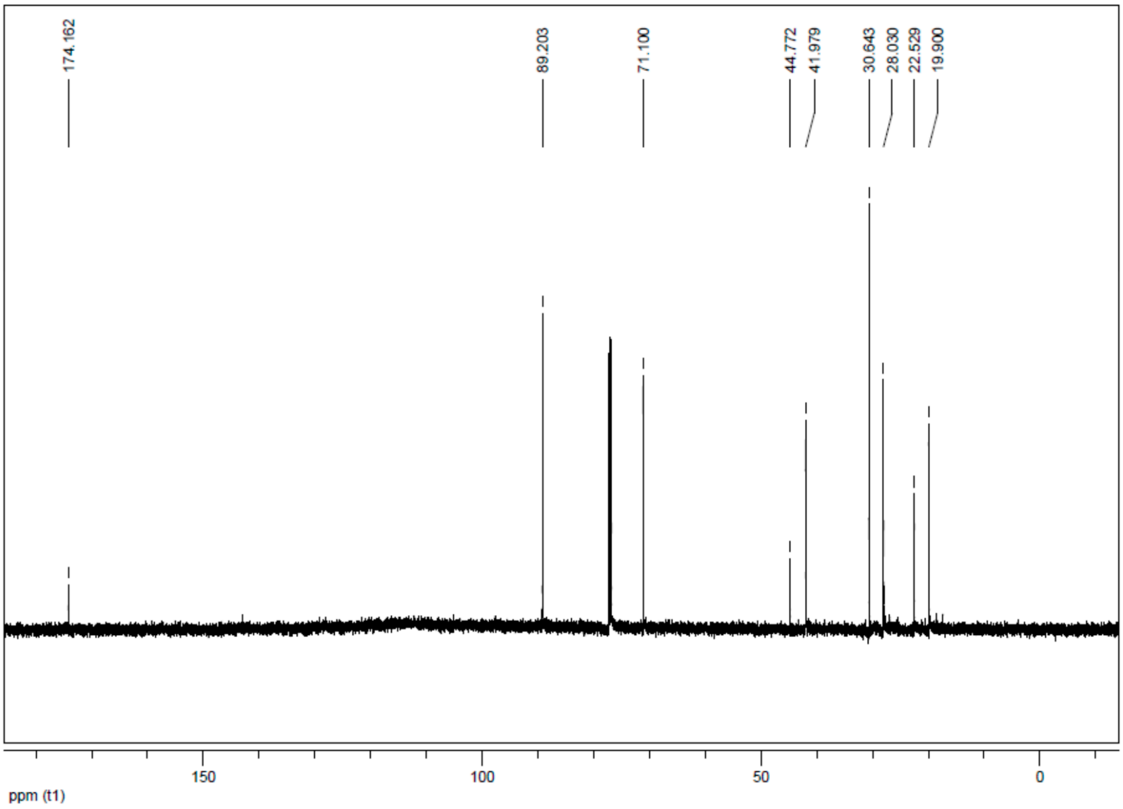

Figure S46. ^{13}C-NMR (151 MHz, $CDCl_3$) spectrum of hydroxy-iodolactone **10**.

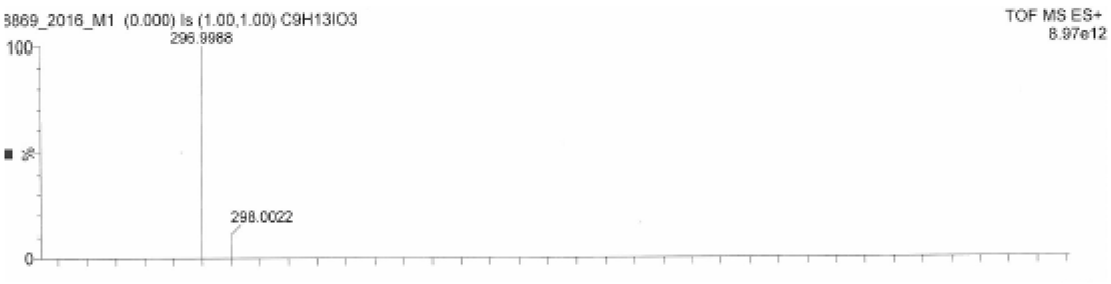

Figure S47. HRMS spectrum of hydroxy-iodolactone **10**.

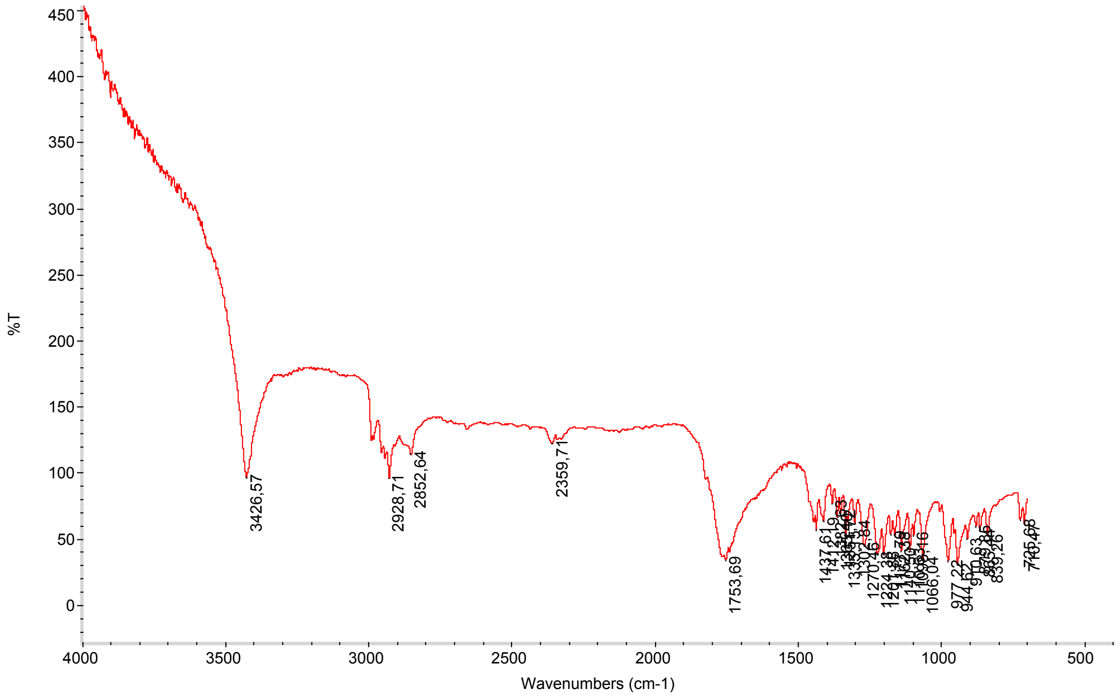

Figure S48. IR spectrum of hydroxy-iodolactone **10**.

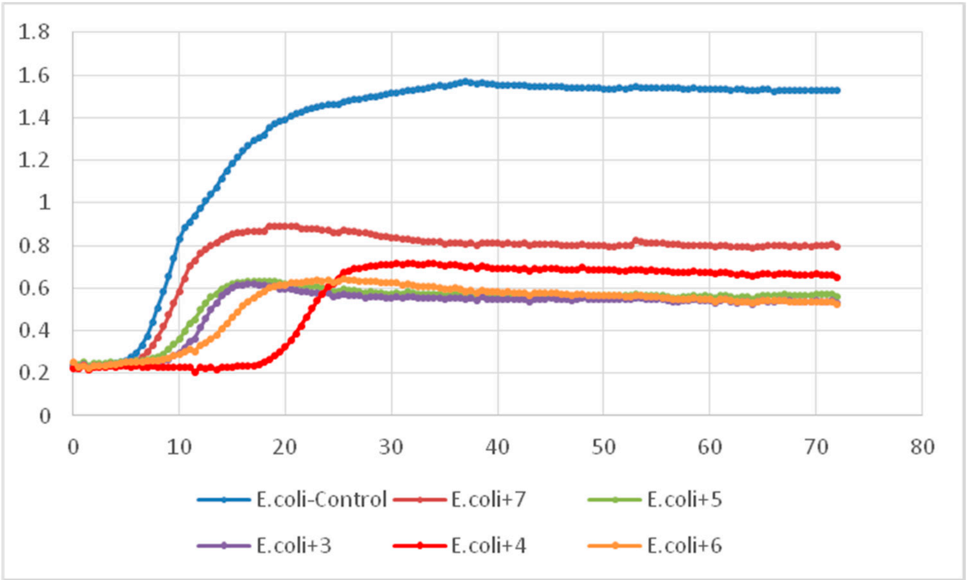

Figure S49. The assessment of the effects of the lactones **3–6** on the growth of *E. coli*.

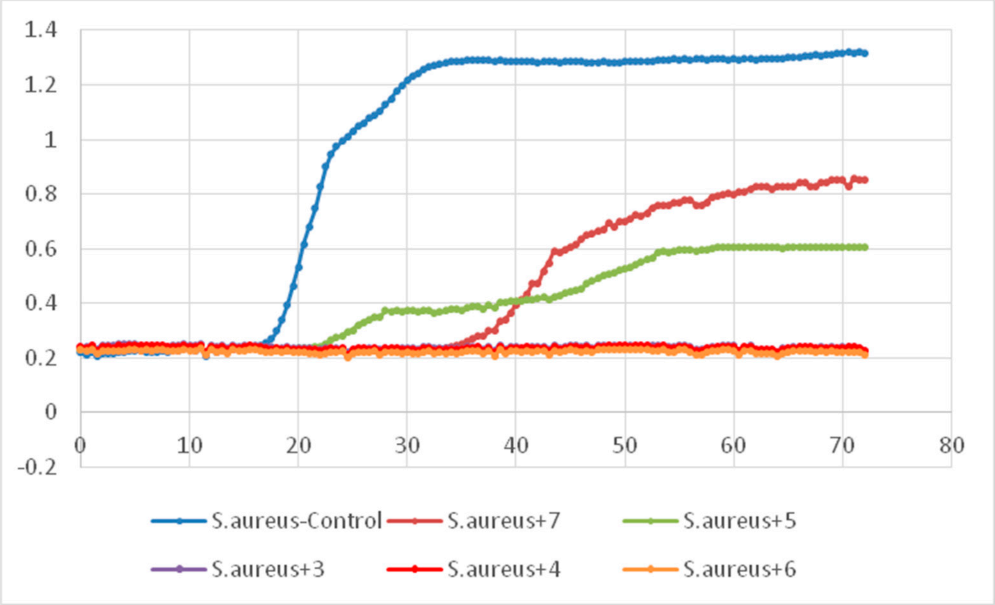

Figure S50. The assessment of the effects of the lactones **3–6** on the growth of *S. aureus*.

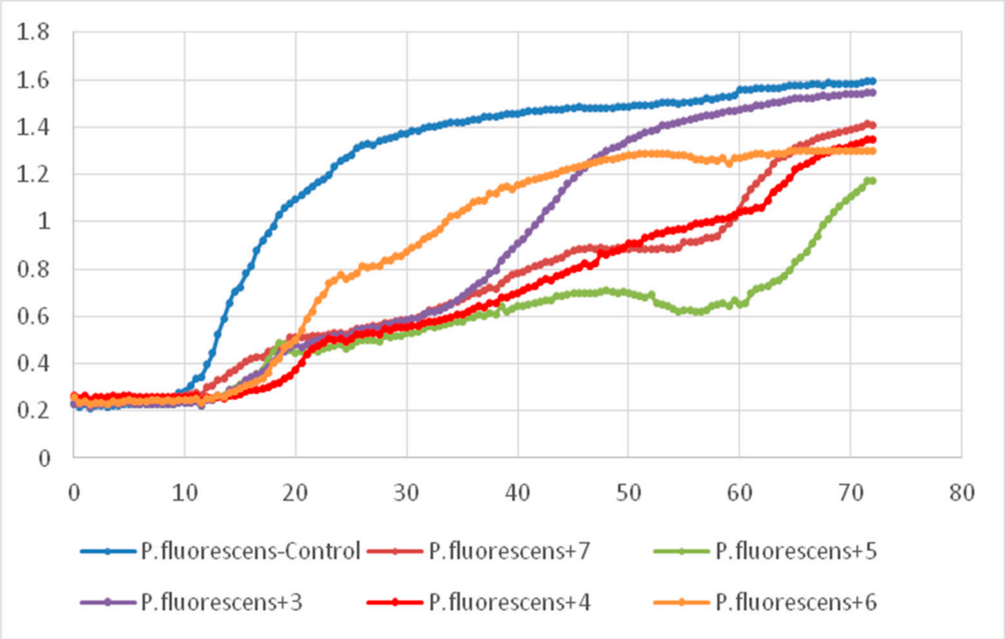

Figure S51. The assessment of the effects of the lactones **3–6** on the growth of *P. fluorescens*.

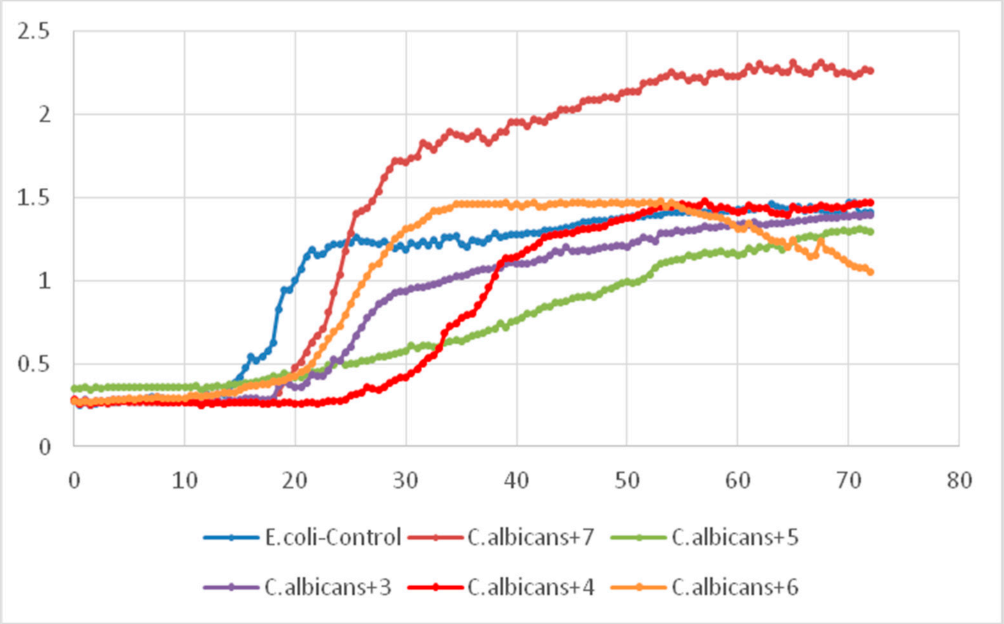

Figure S52. The assessment of the effects of the lactones **3–6** on the growth of *C. albicans*.

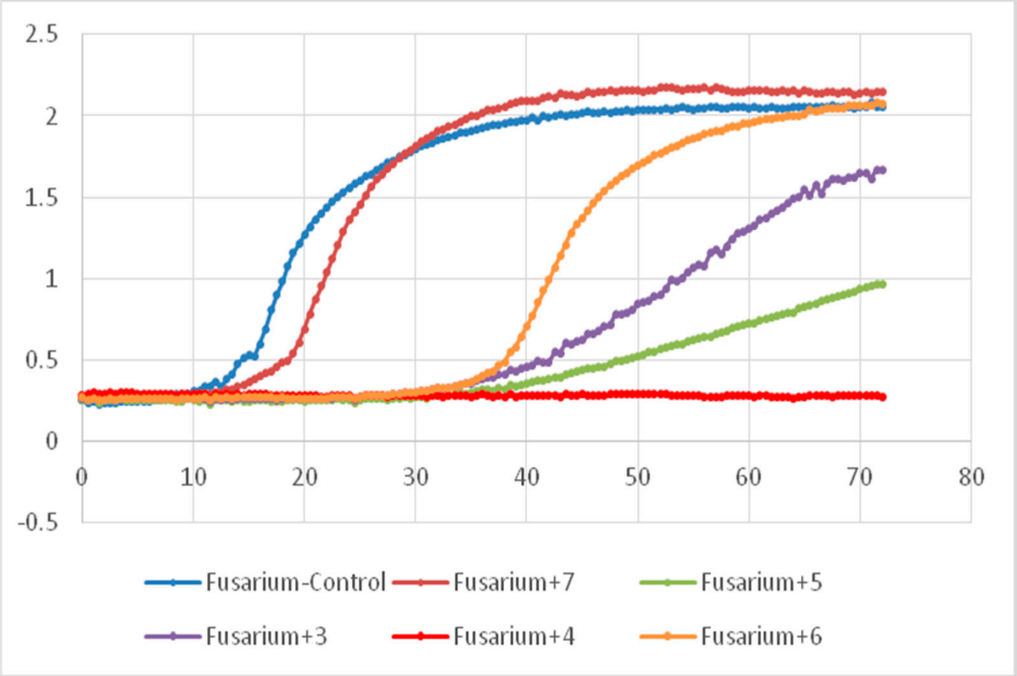

Figure S53. The assessment of the effects of the lactones **3–6** on the growth of *F. avenaceum*.

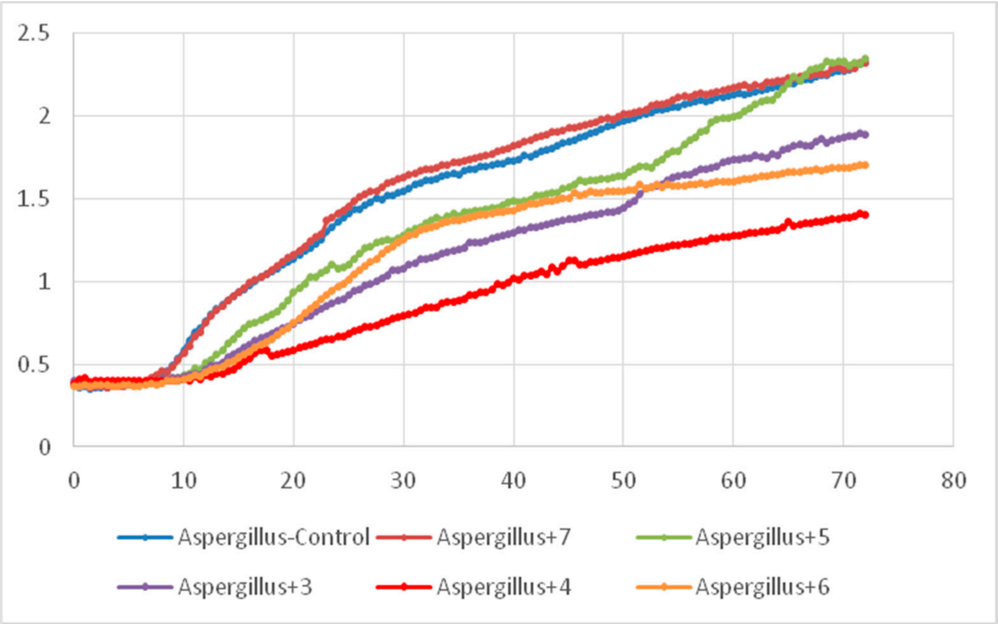

Figure S54. The assessment of the effects of the lactones **3-6** on the growth of *A.niger*

Source of variation	Degrees of freedom	*A niger*	*F avenaceum*	*C.albicans*	*E coli*	*P. fluorescens*	*S. aureus*
Compounds	5	0.471 **	1.507 **	0.530 **	0.455 **	0.083 **	0.589 **
Error	12.000	0.084	0.007	0.005	0.003	0.005	0.006

** significant at $p = 0.01$.

Figure S55. The mean squares analysis of variance (ANOVA) for strains.

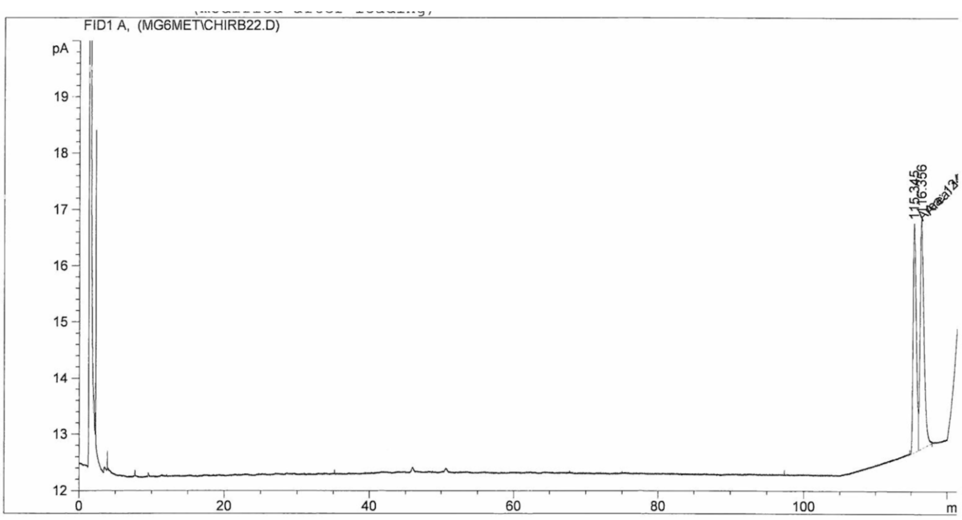

Signal 1: FID1 A,

Peak #	RetTime [min]	Type	Width [min]	Area [pA*s]	Height [pA]	Area %
1	115.345	MM	0.5069	124.18555	4.08300	44.63252
2	116.356	MM	0.6220	154.05449	4.12780	55.36748

Figure S56. Chiral chromatogram of lactone **7** obtained from substrate **3** by *F. equiseti* AM2.

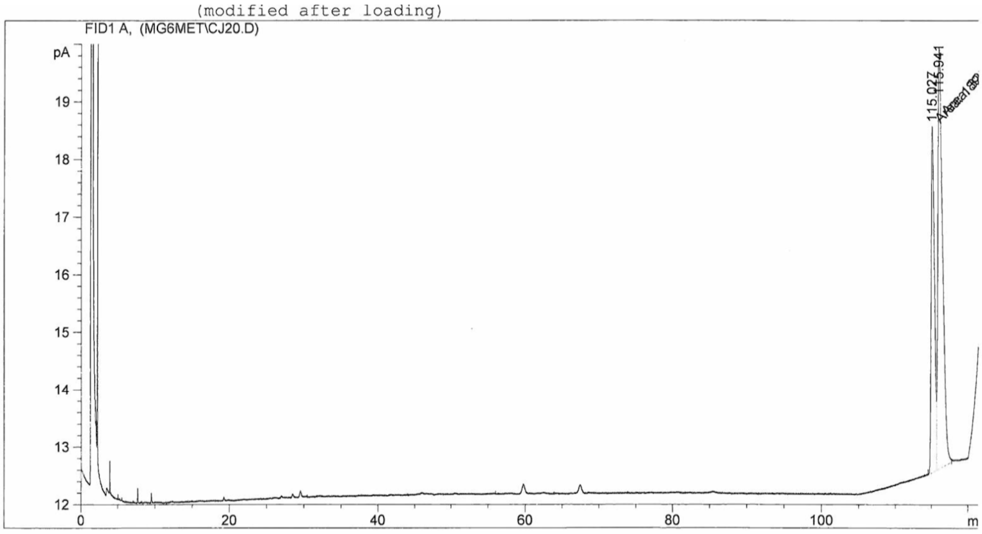

Signal 1: FID1 A,

Peak #	RetTime [min]	Type	Width [min]	Area [pA*s]	Height [pA]	Area %
1	115.027	MM	0.5238	189.44145	6.02790	36.50999
2	115.941	MM	0.7499	329.43427	7.32175	63.49001

Figure S57. Chiral chromatogram of lactone **7** obtained from substrate **4** by *F. equiseti* AM22.

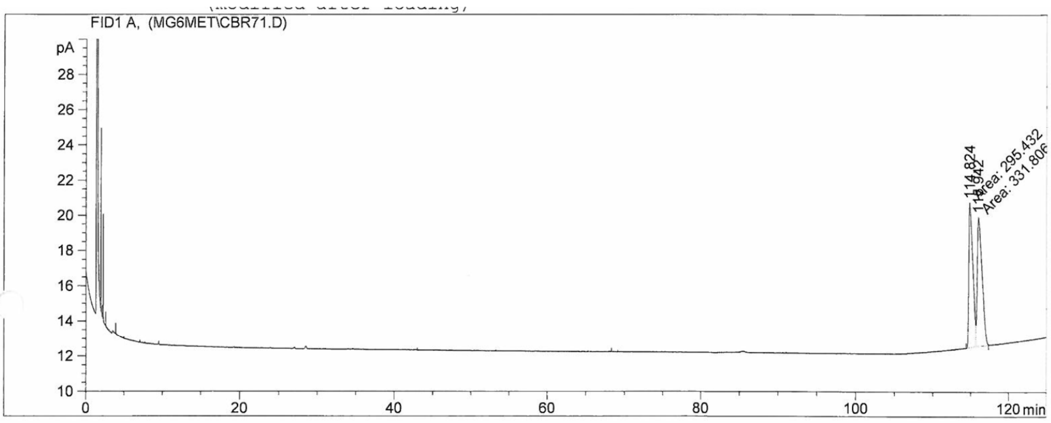

Signal 1: FID1 A,

Peak #	RetTime [min]	Type	Width [min]	Area [pA*s]	Height [pA]	Area %
1	114.824	MM	0.5965	295.43195	8.25409	47.10047
2	115.942	MM	0.7518	331.80588	7.35589	52.89953

Figure S58. Chiral chromatogram of lactone **7** obtained from substrate **3** by *Y. lipolytica* AM71.

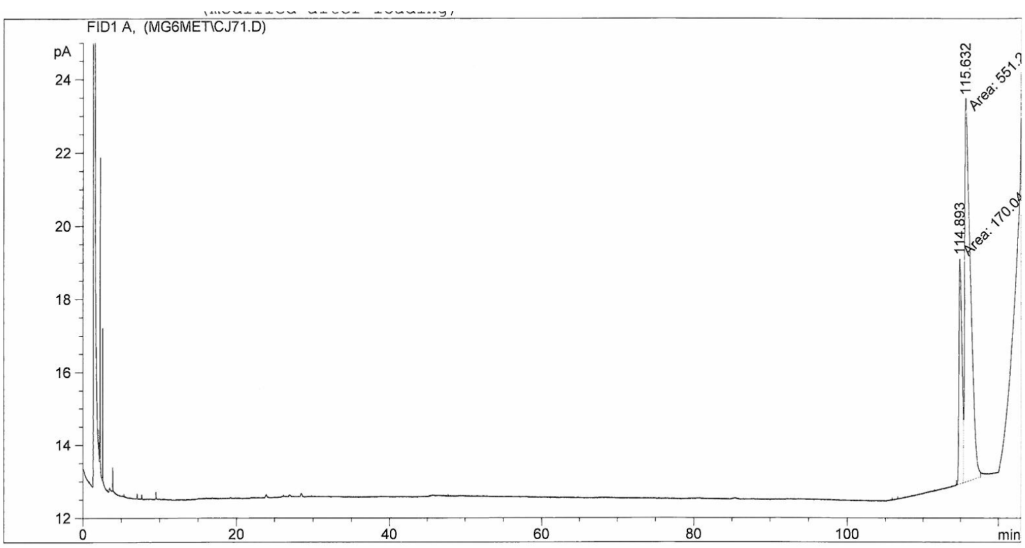

Signal 1: FID1 A,

Peak #	RetTime [min]	Type	Width [min]	Area [pA*s]	Height [pA]	Area %
1	114.893	MM	0.4598	170.04428	6.16433	23.57618
2	115.632	MM	0.8746	551.21027	10.50374	76.42382

Figure S59. Chiral chromatogram of lactone **7** obtained from substrate **4** by *Y. lipolytica* AM71.

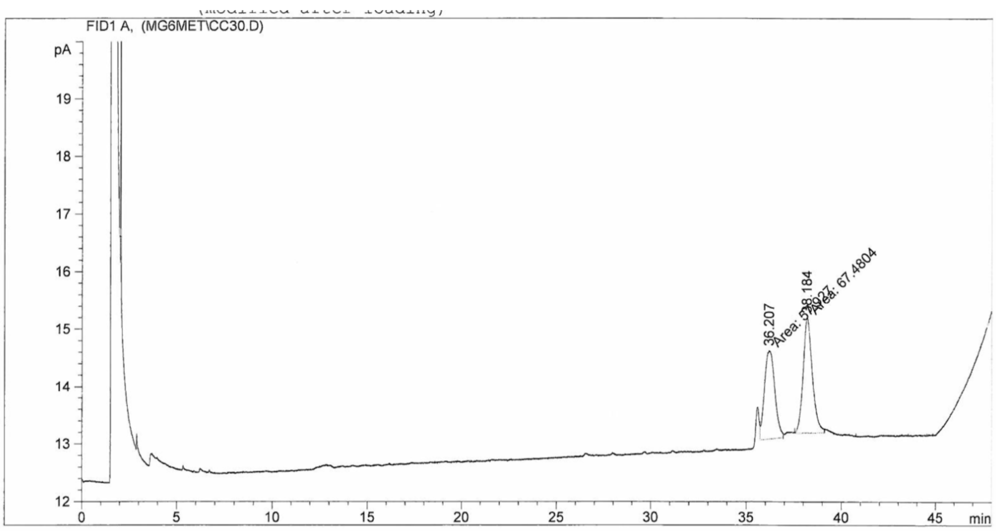

Signal 1: FID1 A,

Peak #	RetTime [min]	Type	Width [min]	Area [pA*s]	Height [pA]	Area %
1	36.207	MM	0.6222	57.92698	1.55162	46.19106
2	38.184	MM	0.5583	67.48038	2.01454	53.80894

Figure S60. Chiral chromatogram of lactone **8** obtained from substrate **2** by *P. vermiculatum* AM30.

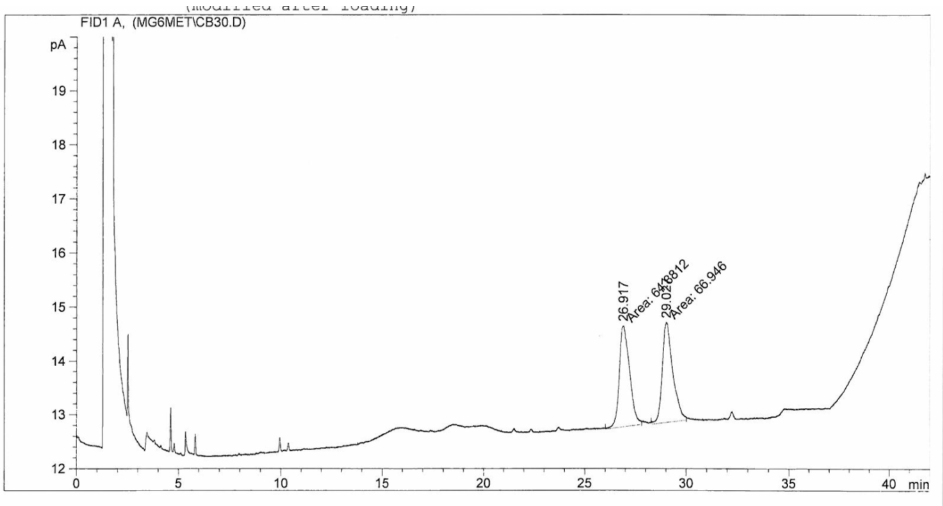

Signal 1: FID1 A,

Peak #	RetTime [min]	Type	Width [min]	Area [pA*s]	Height [pA]	Area %
1	26.917	MM	0.5738	64.88123	1.88450	49.21686
2	29.027	MM	0.5972	66.94600	1.86839	50.78314

Figure S61. Chiral chromatogram of lactone **9** obtained from substrate **3** by *P. vermiculatum* AM30.

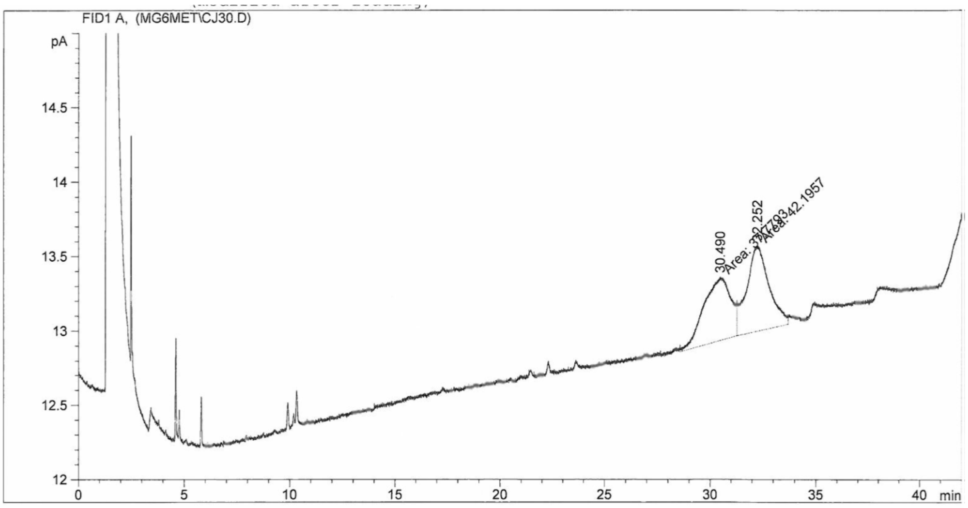

Signal 1: FID1 A,

Peak #	RetTime [min]	Type	Width [min]	Area [pA*s]	Height [pA]	Area %
1	30.490	MM	1.4778	37.77927	4.26075e-1	47.23886
2	32.252	MM	1.2231	42.19572	5.74972e-1	52.76114

Figure S62. Chiral chromatogram of lactone **10** obtained from substrate **4** by *P. vermiculatum* AM30.
